# Persistence, Isolation and Diversification of a Naturally Fragmented Species in Local Refugia: The Case of *Hydromantes strinatii*

**DOI:** 10.1371/journal.pone.0131298

**Published:** 2015-06-24

**Authors:** Roberta Cimmaruta, Daniela Lucente, Giuseppe Nascetti

**Affiliations:** Dipartimento di Scienze Ecologiche e Biologiche, Università della Tuscia, Viterbo, Italy; Estonian Biocentre, ESTONIA

## Abstract

The study of the European plethodontid salamander *Hydromantes strinatii* using allozyme and mitochondrial markers showed a strong geographical genetic structure. This was likely the outcome of different evolutionary mechanisms leaving their signature despite the effects of the genetic drift due to the low population size typical of this species. Two highly divergent clades were identified in the eastern and central-western part of the range, with further geographic sub-structure. Nuclear and mitochondrial markers substantially recovered the same population groups but were conflicting in reconstructing their relationships. This apparent incongruence highlighted the action of different mechanisms such as secondary contacts and incomplete lineage sorting in originating the observed genetic variation. The troglophilic habit of this species provided the opportunity to show the importance of caves as local refugia in maintaining the genetic diversity through the persistence of local populations. Accordingly, high nucleotide and haplotype diversity, strong geographic genetic structuring and lack of expansion were evidenced. This signature was found in the populations from the Ligurian and Maritime Alps, in agreement with the complex orography and paleoclimatic history of this Mediterranean hotspot.

## Introduction

Cave species are considered particularly fragile and endangered due to their strict ecological requirements, making them poorly able to face climatic variation. Also, their strong association with underground retreats constrains their populations to low size and poor genetic diversity [1]. However, some recent data started to overturn this view, especially for troglophilic animals that are able to exploit both subterranean and epigean habitats. These features could make troglophilic species more resistant to climate changes than surface species since they have the possibility to draw back into local retreats under unfavourable external conditions and wait for better climate while sheltered. This hypothesis has been fuelled by recent case studies showing similar patterns of persistence in isolated troglophilic species [2] but also in surface species linked to patchy and heterogeneous habitats, thus pointing out the relevance of localized refugia in warranting the persistence of species through time [3–5]. High genetic diversity coupled with high genetic structuring, minimal population expansion and the persistence of local populations even in the presence of unfavourable Pleistocenic climatic conditions, are among the features observed. This finding has fostered the idea that localized refugia may have had an important role, besides the well known role played by macrorefugia, in assisting species persistence and diversification through Plio-Pleistocenic climate changes, especially in areas affected by deep changes but left free from ice cover [6–9].

The European plethodontid salamanders genus *Hydromantes* are perfect candidates for these kind of studies. Their biological and ecological features would suggest species fragility: *Hydromantes* are lungless thus rely on cutaneous respiration and need steady moist and fresh environments to avoid dehydration and respiratory insufficiency. Moreover these species lack an aquatic larval stage since the metamorphosis is completed within the egg, requiring moist, fresh and steady habitats where females can attend to their eggs until hatching occurs [10–13]. For these reasons there is a strong association of *Hydromantes* with underground retreats, such as caves and fissure systems, that are able to warrant the environmental conditions they need during unfavourable climatic periods such as Mediterranean summer. At the same time, European *Hydromantes* are generally characterized by old lineages and high genetic variation suggesting persistent and stable populations despite their very small size [10,14–16].

With the aim of explaining the apparently contrasting features of European plethodontids in the light of historical climate changes and localized retreats, we analyzed the genetic structure and phylogeography of *H*. *strinatii*. Among the eight species of *Hydromantes* [17,18], *H*. *strinatii* is particularly suited for this type of study since the central and western part of its range is in the Ligurian and Maritime Alps, between Italy and southern France. This area is characterized by a very complex orography, including karst areas, and by a peculiar paleoclimatic history. The ice sheet covering the Western Alps during Quaternary glaciations showed a patchy distribution in the coastal zones and many peripheral areas remained free from ice. These refugial micro-areas now host a strikingly high species richness and endemism concentration of plant species [19–21] and constitute a zone of coexistence of in situ differentiated lineages in animals [22–24].

In this work, we used partial sequences of the mitochondrial genes cytochrome-b (cytb) and NADH-dehydrogenase sub-unit 2 (ND2) and allozyme analysis to study the genetic structure and the phylogeography of *H*. *strinatii*. This allowed for the disentangling of the different evolutionary processes leading to the extant pattern of diversification in *H*. *strinatii*. We verified whether persistence in localized refugia contributed to the present genetic diversity of the species leaving the expected signature of high haplotypic and nucleotidic diversity associated to strong geographic structuring and lack of expansion. Finally, we tried to assess whether the distinctive topography and paleoclimatic history of the Ligurian and Maritime Alps was relevant in determining patterns and processes acting on *H*. *strinatii* by comparing the pattern of genetic diversity observed in the populations from this central-western part of the range vs. those observed in the easternmost populations, living in a more homogeneous environment.

## Materials and Methods

### Ethics statement

The specimens of *H*. *strinatii* we studied belong to the collection of the Zoological Museum “La Specola” of the University of Florence (Italy) and were kindly provided by prof. Benedetto Lanza. Some specimens from a few localities were sampled on purpose under the licenses required by the corresponding authorities.

The permits of collecting, mark, collect tissues and release specimens was given by the Italian Ministry of the Environment (protocols DPN2009/0026530 and 0042634PNM07/08/13) according to the law DPR 357/97. The permits broadly allowed the collection of biological tissues without mentioning any protocol to be followed. No approval of ethics committee was required by both the Italian law and the EU directives on the use of animals for scientific purposes in force during the study period (Legislative Decree 116/92 and EU Directive 609/86).

For tissue sampling salamanders were captured manually and a small piece of about 2–3 mm was cut from the tail tip after keeping the tail on an instant-ice pack for about one minute. No anaesthetics were used to avoid possible mortality due to the high sensitivity of *Hydromantes* skin. Tissue samples were immediately frozen in a portable liquid nitrogen storable tank and sampled individuals were then released. No individuals were brought to the laboratory or sacrificed.

### Sampling and laboratory procedures

**Fig 1:**
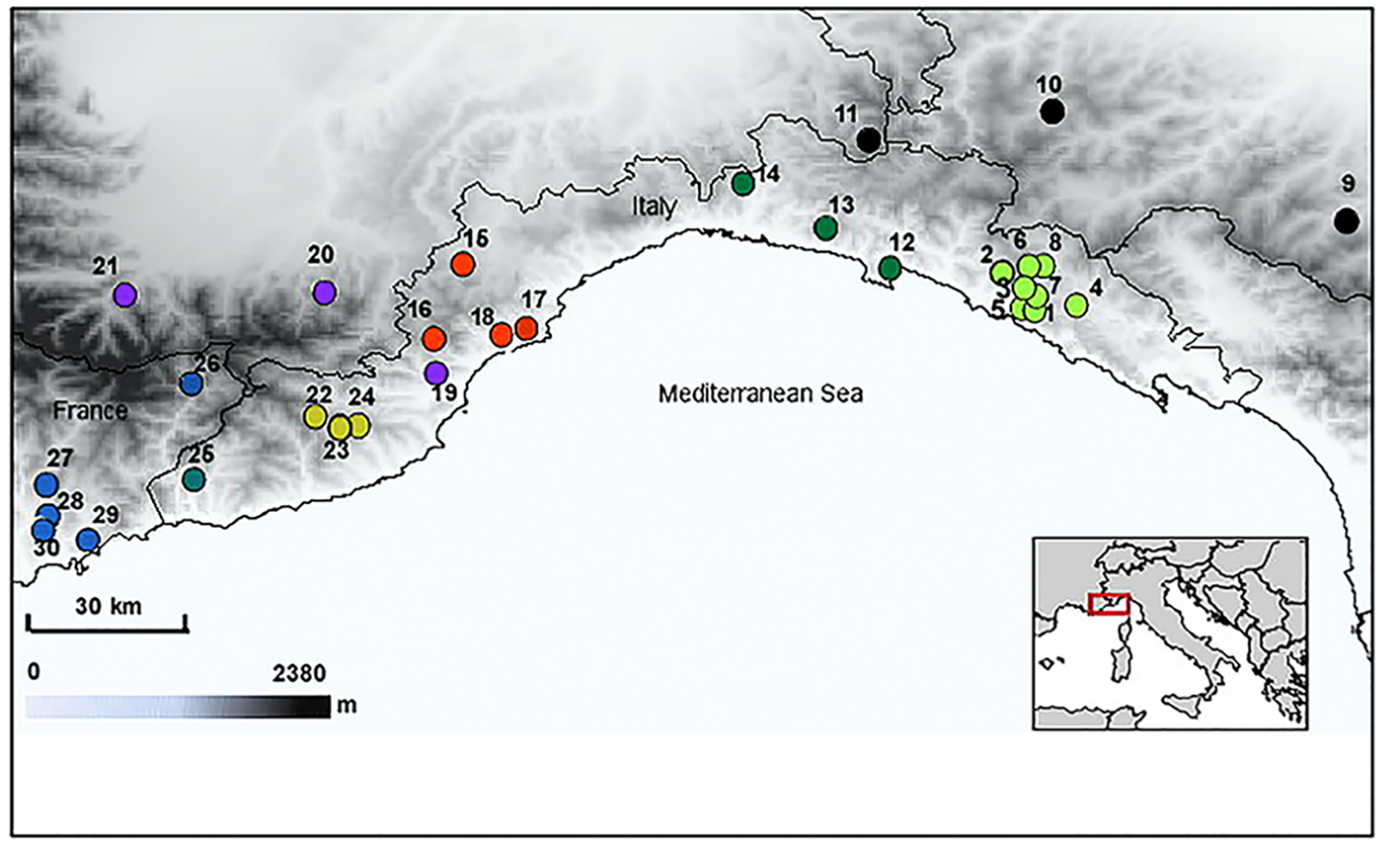
Distribution map of the 30 sampling sites of *Hydromantes strinatii*. Colours denote the seven sub-clades recovered by phylogenetic analyses, with the exception of black populations that were included in allozyme analysis only.

An overall number of 385 specimens of *H*. *strinatii* were analysed from 30 sites distributed throughout the species range (Table 1, Fig 1). Standard horizontal starch gel electrophoresis was carried out for all samples using 33 putative allozyme loci (S1 Table) and using the laboratory procedures described in detail in [11].

**Table 1:** Sampling localities.

n°	Locality	Code	Latitude	Longitude	N_nc_	N_mt_	mtDNA haplotypes
**1**	Pietra Vasca Mt.—Genoa, Ligury	SBR*	44°15'35.00"	9°30'43.00"	34	5	hSt1 (5)
**2**	Groppi Mt.—Genoa, Ligury	SBC*	44°15'18.00"	9°32'33.00"	8	4	hSt1 (2), hSt3 (2)
**3**	Environs of Carro—Genoa, Ligury	SSM*	44°16'0.00"	9°38'23.00"	10	6	hSt1 (5), hSt4 (1)
**4**	Pù Mt.—Genoa, Ligury	SCG*	44°17'11.00"	9°30'49.00"	8	3	hSt1 (3)
**5**	Cassagna—Genoa, Ligury	SCA	44°20'16.00"	9°28'1.00"	16	14	hSt1 (12), hSt2 (1), hSt5 (1)
**6**	Cave "Tana de Strie"—La Spezia, Ligury	STS*	44°19'28"	9°31'9.00"	17	5	hSt1 (5)
**7**	Cave "Tana da Cruxetta"—La Spezia, Ligury	SCR*	44°20'9.00"	9°31'39.00"	8	8	hSt1 (8)
**8**	Maissana and SW slope of Baralucco Mt.—La Spezia, Ligury	SBN	44°21'9.00"	9°31'47.00"	15	14	hSt1 (14)
**9**	Codolo—Massa and Carrara, Tuscany	SCO*	44°22'05''	9°50'26''	11	-	-
**10**	Bardi—Parma, Emilia—Romagna	SBD	44°42'28''	09°41'52''	7	-	-
**11**	Carrega Ligure—Alessandria, Piedmont	SAL*	44°36'33.34"	9°10'31.43	10	-	-
**12**	Rapallo—Genoa, Ligury	SRA*	44°20'52.00"	9°12'25.00"	14	1	hSt6 (1)
**13**	Bargagli—Genoa, Ligury	SBA*	44°26'6.00"	9° 3'27.00"	18	15	hSt6 (15)
**14**	Isoverde—Genoa, Ligury	SIS*	44°31'56.00"	8°51'59.00"	24	16	hSt6 (15), hSt7 (1)
**15**	Millesimo—Savona, Ligury	SOR	44°21'22.00"	8°12'52.00"	5	2	hSt8 (2)
**16**	Bardineto—Savona, Ligury	SRI*	44°11'36.00"	8° 8'52.00"	5	1	hSt8 (1)
**17**	Finale Ligure—Savona, Ligury	STA*	44°12'57.00"	8°21'46.00"	17	11	hSt8 (4), hSt9 (5), hSt10 (1), hSt11 (1)
**18**	Gavone—Savona, Ligury	SGA	44°10'34"	8°19'35"	5	-	-
**19**	Toirano—Savona, Ligury	STO*	44° 8'16.00"	8°10'09.00"	29	12	hSt14 (12)
**20**	Rio Roburentello stream—Cuneo, Piedmont	SSL*	44°17'41.00"	7°53'25.00"	10	5	hSt13 (5)
**21**	Roaschia and Rivoera	STR*	44°17'23.00"	7°25'49.00"	12	10	hSt12 (5), hSt13 (5)
**22**	Rezzo—Imperia, Ligury	SRE*	44° 1'21.06"	7°52'21.00"	-	3	hSt15 (1), hSt16 (1), hSt18 (1)
**23**	San Bartolomeo Hill—Imperia, Ligury	SSG*	44° 0'4.00"	7°56'13.00"	18	11	hSt15 (11)
**24**	Cavaronica—Imperia, Ligury	STC*	44° 0'12.00"	7°56'46.00"	14	7	hSt15 (6), hSt17 (1)
**25**	Tenarda—Imperia, Ligury	STE*	43°53'30"	7°35'07"	30	24	hSt19 (10), hSt20 (10), hSt21 (1), hSt22 (2), hSt23 (1)
**26**	Tenda	STN*	44° 5'22.79"	7°35'5.57"	10	4	hSt29 (2), hSt30 (2)
**27**	Luceram	SLU*	43°52'31.00"	7°14'50.00"	4	3	hSt26 (3)
**28**	Peille	SPE*	43°48'25.00"	7°15'10.00"	5	2	hSt25 (1), hSt28 (1)
**29**	Mont Bastide	SMB*	43°44'15.36"	7°21'7.47"	8	6	hSt24 (6)
**30**	Aspremont	SAS*	43°46'48.00"	7°14'31.00"	13	2	hSt27 (2)

Sampling localities with their codes (indicating the species, S = *H*. *strinatii*, and the first two letters from the collecting cave/locality), coordinates, number of individuals analysed using allozymes (N_nc_) and mtDNA markers (N_mt_), and the recovered haplotypes. Samples belonging to the collection of the Zoological Museum “La Specola” of Florence (Italy) are indicated by *, the others were collected in field work.

Total genomic DNA was extracted from the frozen tissue of 194 individuals using the cetyltrimethylammonium bromide (CTAB) protocol [25].

Partial sequences of the mitochondrial genes cytochrome-b (cytb) and NADH dehydrogenase subunit 2 (ND2) were obtained using specifically designed primers: HST-1f (5’-TTTATTGATTTACCAACCCCATCT-3’) and DEB-1r (5’-CAGGGGTGAAGTTTTCTGGAT-3’) for cytb; HND1-f (5’-TCAAGCTTATCCACCGGAAC-3’) and MAT2-r (5’-TTGGTGGTAGTCCGCCTAAG-3’) for ND2. Amplification was performed by polymerase chain reaction (PCR) in a volume of 25 μl containing MgCl2 (2.5 mM), the reaction buffer (1X; Promega), the four dNTPs (0.2 mM each), the two primers (0.2 μM each), the enzyme Taq polymerase (2 U; Promega) and 2 μl of DNA template. PCR reactions were carried out in a Hain Lifescience Q-Sat 24 thermal cycler using the following program: denaturation at 94°C for 5 minutes followed by 35 cycles at 94°C for 30 s, 53°C for 1 min, 72°C for 1 min and a final elongation step at 72°C for 10 min.

Purification and sequencing reactions were outsourced to Macrogen Inc. (www.macrogen.com).

Electropherograms were independently checked by eye by two of the Authors using Chromas v.1.6 (Technelysium Pty Ltd) and sequences were then aligned using the software ClustalX v.1.83 [26].

### Mitochondrial data analysis

#### Phylogenetic analysis

In order to reconstruct the phylogenetic relationships between mitochondrial lineages, we conducted maximum likelihood (ML) and Bayesian inference (BI) analyses for the combined dataset of cytb and ND2 genes, and applying a partition strategy by genes in both cases. Sequences of both cytb and ND2 genes were obtained from Sardinian species *H*. *supramontis* and *H*. *imperialis* and were used as outgroups (GenBank accession n. KJ834013-14 and KJ834057-58)

We computed ML analysis in RAxML GUI v.1.3 [27] using a GTRGAMMA model and setting partitions by gene. The ‘ML + thorough bootstrap’ option, with one thousand bootstrap replicates and ten independent searches was implemented and a 50% majority-rule consensus tree was computed. Bayesian inference was carried out using MrBayes v.3.2.2 [28] and specifying different evolutionary models for each gene. When the outgroup was excluded, the following optimal models of sequence evolution were selected with jModelTest v.2.1.2 [29] under a Bayesian Information Criterion (BIC) and starting with a ML optimized tree: TIM3+I for the cytb gene and TrN+I for the ND2 gene. We ran three ‘heated’ and one ‘cold’ chain for 2 million generations, and sampled every 100 generations discarding 25% of the total sampled trees from the beginning of the chain as burn-in. Standard deviations of split frequencies among chains resulted in less than 0.01, and potential scales reduction factors (PSRF) for all parameters were close to one indicating that convergence was reached. We used Tracer v1.6.0 to check for an acceptable number of independent samples [30].

The distance-based least squares (LS) method [31] implemented in DAMBE 5.3.109 [32] was used to estimate the Time to the Most Recent Common Ancestor (TMRCA) after determining that the evolutionary rate was constant through a likelihood ratio test. The analysis was carried out on the topology of the Bayesian tree for the concatenated data set using the ‘MLCompositeTN93’ genetic distance. According to the literature, all the Authors agree in dating back the split between eastern Sardinian and mainland *Hydromantes* lineages to the Messinian salinity crisis regardless of the markers used in their studies [15,17]. Therefore, the analyses were conducted by constraining the split node between mainland *Hydromantes* species (represented by *H*. *strinatii*) and Eastern Sardinian species (represented by *H*. *supramontis* and *H*. *imperialis*) at 5.33 ma, which is the end of the Messinian salinity crisis (5.96–5.33 ma) [33,34]. The standard deviation of the time estimates was calculated by bootstrap re-sampling with 1000 pseudoreplicates. Construction of a haplotype network was carried out by a median-joining (MJ) algorithm [35] as implemented in the software Network v.4.6.1.2 (http://www.fluxus-engineering.com) under Greedy FHP criterion [36] and default parameter values. Genetic distances between the haplo-groups identified were computed in MEGA [37] under the Kimura two-parameter model (K2P) [38].

#### Population genetic structure and demographic analysis

To investigate population genetic structure, we first ran our data in SAMOVA v.1.0 (Spatial Analysis of Molecular Variance; [39]) testing K values from 2 to 20 by performing 100 simulated annealing processes. Additionally, we carried out the identification of groups under a Bayesian clusterization approach using the software BAPS v.6.0 [40–42]. We implemented a genetic mixture analyses using the non spatial clustering option and ran five replicates for each K ranging between 2 to 20.

Once the best number of groups was selected according to both SAMOVA and BAPS, all the subsequent analyses were carried out on each detected population group.

Haplotype (h) and nucleotide (π) diversity as well as the gene flow parameter Nm were estimated for each cluster using DNAsp v5.10 [43].

A hierarchical analysis of molecular variance (AMOVA; [44]) was carried out in Arlequin v.3.5 [45] using a pairwise F_st_ distance matrix and running 1000 permutations.

Tajima’s D [46], Fu’s Fs [47] and Ramos-Onsins & Rozas’ R2 [48] statistical neutrality tests were applied to each group to detect possible historical population growth. Also, we examined the distribution of the number of differences between pairs of haplotypes by a mismatch analysis [49] and evaluating both the sum of square deviations (SSD) [50] and Harpending’s raggedness index (RI) [51] for each group. Tajima’s D, Fu’s Fs, and the mismatch distribution were computed in Arlequin v.3.5 [45], while we used DNAsp v.5 for R2 computations [43].

To verify if the population genetic structure conformed to an isolation by distance model (IBD), a Mantel test was executed in Arlequin v.3.5 [45]. A genetic F_st_ distance matrix was performed against a geographic distance matrix (with distances expressed in km) using 10000 permutations. Computations were done for each single group and for the whole population set.

### Allozyme data analysis

Allozyme allele frequencies, conformity to Hardy-Weinberg equilibrium and linkage-disequilibria were calculated using the software Genepop 4.2 [52] and testing for departures from expectations using Fisher’s method. The parameters of genetic variability per population were estimated using the software Genetix [53]: mean number of alleles (N_a_), observed heterozygosity (H_o_), expected heterozygosisty (H_e_), percentage of polymorphic loci under 99% criterion (P_99_). The softwares Genodive 2.0 [54] and FSTAT 2.9.3.2 [55] were used to estimate the effective number of alleles (N_E_) and the allelic richness (A_R_), respectively.

The partitioning of genetic diversity was analyzed for variable loci using the Fixation index F_st_ as implemented in Genepop 4.2 [52]. The significance of pairwise F_st_ values was tested using Fisher’s method followed by Bonferroni correction [56].

A Principal Component Analysis was carried out based on a matrix of covariance of allele frequencies as implemented in Genodive 2.0 [54], and the significance of the population differentiation represented by the PCA-axes was tested by 100000 permutations.

The identification of population groups was obtained using BAPS v.6.0 [40,57], setting the non-spatial clustering option and running 5 replicates for each K value between 2 and 20. Also, a Bayesian model-based algorithm was used to assign individuals to genetically homogeneous groups, as implemented in STRUCTURE v.2.3.4 [58]. We used an admixture ancestry model assuming correlated allele frequencies and different values of F_st_ for different subpopulations due to the high levels of natural fragmentation of *Hydromantes* populations. Priors were set choosing sampling location information (LOCPRIOR) and using both the mean value of F_st_ and the associated standard deviation obtained by previous calculations. Length of the burn-in period was of 100000 replicates followed by 100000 Markov chains Monte Carlo (MCMC) replicates, and fifteen runs were conducted for each K ranging from 2 to 12. The Evanno algorithm [59], as implemented in Structure Harvester v.0.6.94 [60], was used to select the optimal K based on both the log probability of the data and the inferred ∆K. The independent runs relative to the optimal K value were aligned with CLUMPP v.1.1.2 [61] under the Greedy algorithm. The results were graphically displayed by DISTRUCT v.1.1 [62].

An analysis of molecular variance was carried out assuming a stepwise model as implemented in Genodive 2.0 [54] and partitioning populations according to the K = 5 groups recovered by STRUCTURE.

A Mantel test was performed in Arlequin v.3.5 [45] contrasting a F_st_ distance matrix vs. a geographic distance matrix (km). The IBD hypothesis was tested for the whole population set, for the two main mitochondrial clades and for the K groups recovered by STRUCTURE.

## Results

### Mitochondrial data analysis

#### Sequence variation

The 687-bp fragment of cyt-b sequenced had 59 variable positions (5 singletons) 54 of which were parsimony informative, and provided 24 different haplotypes over the 194 specimens scored. The obtained sequences have been deposited in GenBank (Accession n: KJ834015-38). Nucleotide percentages were: T 33.9%, C 22.0%, A 30.4%, and G 13.7%. The estimated nucleotide diversity (π) had a mean value of 0.028.

The 702 bp fragment from ND2 gene had 54 variable positions (1 singleton), 53 of which were parsimony informative. The 194 specimens scored provided 18 haplotypes, available in GenBank (Accession n: KJ834039-56). Nucleotide percentages were: T 31.2%, C 23.2%, A 35.6% and G 10.0%. The estimated nucleotide diversity (π) had a mean value of 0.025.

The alignment of the concatenated sequences was of 1389 bp, and provided an overall number of 30 haplotypes, as listed in Table 1. The estimated values of nucleotide (π) and haplotype (h) diversity were 0.026 and 0.87, respectively. The genetic divergence calculated according to Kimura 2 Parameter (K2P) was 0.028 (±0.003).

Each haplotype was recovered in a single or a few geographically very close localities (Table 1).

#### Phylogenetic analysis

**Fig 2:**
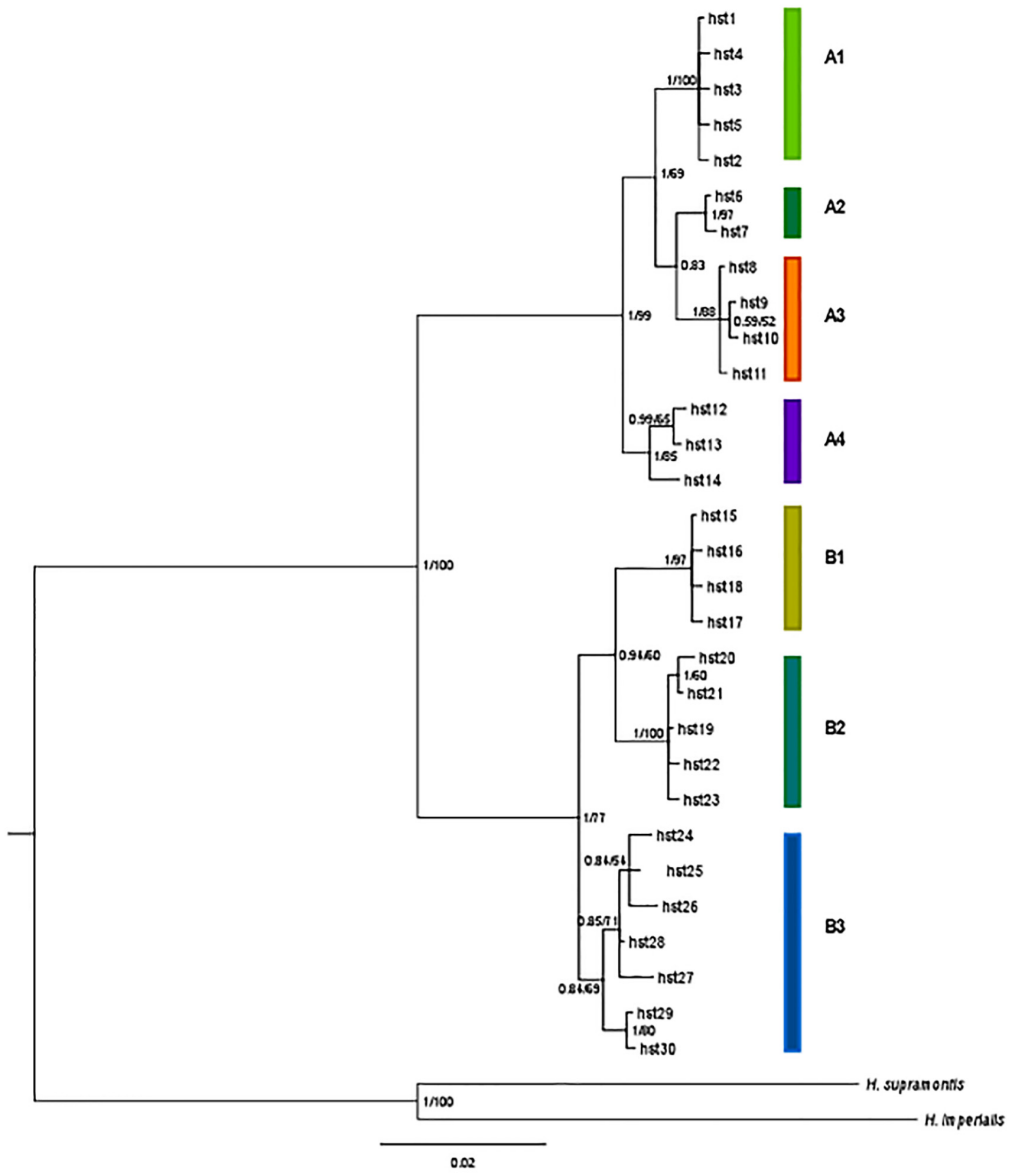
Phylogenetic relationships between mtDNA haplotypes. The tree shows the results for Bayesian Inference (BI) and Maximum Likelihood (ML) analyses. Posterior probability and bootstrap values are reported on each tree node. Both phylogenetic reconstructions showed the occurrence of two main clades A and B, subdivided respectively into four (A1, A2, A3, A4) and three (B1, B2, B3) sub-clades.

**Fig 3:**
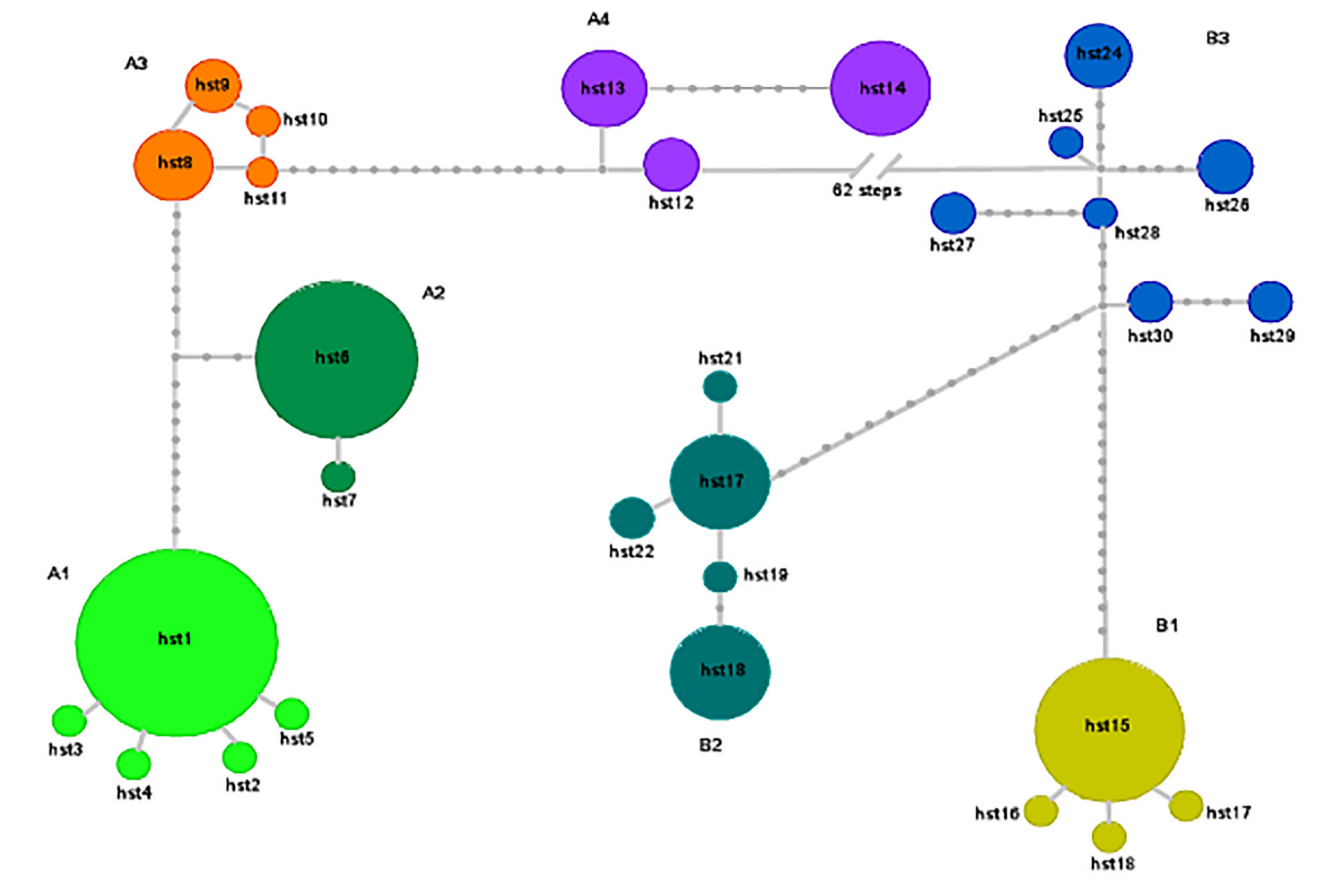
Median joining haplotype network based on the 194 mitochondrial concatenated sequences of cytb and ND2 genes. The size of the circles is proportional to the frequency of each haplotype. Haplotypes are connected by distances proportional to mutational steps with the small grey circles representing missing haplotypes. The seven haplogroups are congruent to the seven sub-clades recovered by phylogenetic analysis; the two main clades A and B are separated by 62 mutational steps.

All the phylogenetic reconstructions agreed in showing two well supported reciprocally monophyletic groups, including the haplotypes recovered in the eastern and western part of the range, respectively: the eastern clade (Clade A) included the haplotypes recovered from samples 1 to 21 and the western clade (Clade B) contained those from samples 22 to 30 (Fig 2). These two clades received a high support for both ML and BI analyses (Fig 2). Clade A was further subdivided into four geographically distinct sub-clades including the haplotypes from: the easternmost part of the range (sub-clade A1, samples 1–11), the “Rapallo area” (sub-clade A2, samples 12–14), the “Finalese” region (sub-clade A3, samples 15–18), and the “Roburent” region (sub-clade A4, samples 19–21). Clade B was subdivided into three sub-clades each including the haplotypes from a geographic area: “San Bartolomeo Hills” (B1, samples 22–24), “Tenarda” area in the Ligurian Alps (B2, sample 25), and the French Maritime Alps (B3, samples 26–30). The outcome of the median-joining network calculation provided further support for the results of the tree-building methods. We recovered the same two main phylogroups (A and B) separated from a total of 62 steps (Fig 3). Within each phylogroup, the haplotypes were structured in seven geographically distinct haplogroups connected by 7 to 17 mutational steps and completely congruent with the seven sub-clades recovered in the tree-based analyses. Sub-clade A1 displayed a star-like pattern, with the haplotype hst1 found at a high frequency in all the samples and representing 91.5% of its haplotypic diversity. In the sub-clade B3, by grouping the haplotypes from the French Maritime Alps, each population was characterized by its own exclusive haplotype(s), and the haplotypes recovered from Tenda (STN, 26) showed an intermediate position between the sub-clade B2 (Tenarda, STE, sample 25) and the other haplotypes from the French Maritime Alps (Fig 3).

The estimates of K2P distance between the seven lineages gave values comprised between 0.8% and 5.4%. The minimum divergence of 0.8% (p-distance = 0.8%) was recovered between the sub-clades A2 and A3. The values of maximum divergence comprised between 5.0% and 5.4% (p-distance values from 4.7% to 5.2%) were always found in pairwise comparisons between sub- clades A vs. B.

#### Population genetic structure and demographic history

The SAMOVA analysis showed that the best partitioning of genetic variation was achieved with seven clusters: F_ct_ index grew from 0.79 to 0.95 for K between 2 and 7 and then slightly moved between 0.96 and 0.99 for K between 7 and 25 (S1 File). Accordingly, the algorithm implemented in BAPS recovered 7 as the best group membership solution with a posterior probability of 1.00 (S1 File). The 7 population groups inferred were completely coincident with the seven sub-clades recovered with the phylogenetic analyses, confirming the genetic and geographic pattern of differentiation previously shown. The AMOVA analysis was performed on the seven groups recovered by SAMOVA and showed that nearly all the genetic variation was due to the among-group level of variation (95.23%, p < 0.0001), while 3.78% was due to the among-population within-group level and 1.00% to the within-population level (Table 2).

**Table 2:** Analysis of molecular variance (AMOVA).

**Mitochondrial data**				
**Source of variation**	**d.f.**	**Sum of squares**	**Variance components**	**Percentage of variation**
**Among groups**	6	3560.285	21.969	95.23
**Among populations within groups**	19	103.478	0.871	3.78
**Within populations**	168	38.650	0.230	1.00
**Allozyme data**				
**Among groups**	4	52693.036	148.014	46.90
**Among populations within groups**	24	19946.880	48.536	15.40
**Within populations**	356	36576.915	119.263	37.80

Hierarchical subdivision of genetic variance among the population groups identified based on mitochondrial (seven groups) and allozyme data (five groups).

The seven clades had heterogeneous levels of both haplotype and nucleotide diversity with lower values presented by the easternmost sub-clades (A1, A2; π spanning 0.00004 to 0.00012; h ranging between 0.063 and 0.163) and the highest values recorded for the French Maritime Alps clade (B3) with π = 0.00472 and h = 0.846.

Inferred levels of gene flow were very low both between groups (average Nm = 0.02) and within groups (Nm = 0.04–1.93) with the lowest values recorded within sub-clades A4 and B3.

The Mantel test results showed no correlation between genetic and geographic distances within the seven groups detected. However, a pattern of IBD came out when considering the entire species range (r = 0.52; p < 0.0001). A significant correlation was also observed within clade A (r = 0.71; p < 0.0001), while clade B exhibited no IBD (r = 0.22; p < 0.11).

The demographic test statistics and the analysis of the mismatch distribution computed for each of the seven clades showed only weak signs of past demographic expansion (Table 3). Both Tajima’s D and Fu’s Fs gave negative and significant values for the eastern clade A1 and for the S. Bartolomeo clade B1. However, the most sensitive R2 test did not result in any significant value. On the contrary, the mismatch distribution showed that the frequencies of pairwise haplotype differences expected under the assumption of a sudden demographic expansion and those observed from the data were similar for all the clades except B3, so the parametric bootstrap approach did not reject the sudden expansion model for the other 6 clades (A1, A2, A3, A4, B1, B2). However, the curves obtained from observed vs. expected differences were graphically coincidental only for clades A1, A2 and B1. Overall, the values obtained testing the demographic history of *H*. *strinatii* provided weak and sometimes contrasting signals coherently supporting a past expansion only for clades A1 and B1.

**Table 3:** Results of genetic diversity estimates, neutrality tests and mismatch distribution for mitochondrial data.

Sub-Clade							Demographic expansion		Spatial expansion	
	N	Hd	π	Tajima's D test	Fu's FS test	R2	SSD	RI	SSD	RI
mtA1 (Eastern)	59	0.163	0.00012	-1.762	-5.228	0.0555	0.00068	0.48452	0.00068	0.48452
*p*	* *	* *	* *	*0*.*005*	*0*.*001*	*0*.*158*	*0*.*340*	*0*.*70*	*0*.*68*	*0*.*40*
mtA2 (Rapallo)	32	0.063	0.00004	-1.14244	-1.264	0.1613	0.00001	0.76953	0.00001	0.76953
*p*	* *	* *	* *	*0*.*134*	*0*.*003*	*0*.*677*	*0*.*210*	*0*.*83*	*0*.*18*	*0*.*85*
mtA3 (Finalese)	14	0.659	0.00057	0.700	-0.929	0.1800	0.0289	0.20879	0.02892	0.20879
*p*	* *	* *	* *	*0*.*802*	*0*.*135*	*0*.*630*	*0*.*110*	*0*.*11*	*0*.*06*	*0*.*10*
mtA4 (Roburent)	24	0.67	0.00128	0.944	0.636	0.1739	0.0735	0.21333	0.05312	0.21333
*p*	* *	* *	* *	*0*.*86*	*0*.*676*	*0*.*852*	*0*.*20*	*0*.*18*	*0*.*18*	*0*.*47*
mtB1 (S. Bartolomeo)	27	0.655	0.00304	2.566	7.779	0.2343	0.1775	0.45163	0.10843	0.45163
*p*	* *	* *	* *	*0*.*996*	*0*.*995*	*1*.*000*	*0*.*07*	*0*.*00*	*0*.*10*	*0*.*35*
mtB2 (Tenarda)	21	0.271	0.00021	-1.726	-2.819	0.1166	0.0049	0.28143	0.00110	0.28143
*p*	* *	* *	* *	*0*.*019*	*0*	*0*.*170*	*0*.*42*	*0*.*58*	*0*.*48*	*0*.*57*
mtB3 (Ma-ritime Alps)	17	0.846	0.00472	0.430	2.057	0.1615	0.0516	0.12084	0.03707	0.12084
*p*	* *	* *	* *	*0*.*723*	*0*.*839*	*0*.*744*	*0*.*01*	*0*.*01*	*0*.*33*	*0*.*56*

For each of the seven mitochondrial groups identified with phylogenetic analyses are reported: number of individuals analysed (N), haplotype diversity (Hd), nucleotide diversity (π); values of Tajima’s D, Fu’s Fs and R2 tests; sum of squared deviations (SSD) and raggedness index (RI). *p*-values associated to neutrality tests and mismatch distribution statistics are also reported.

The chronogram with the obtained confidence intervals (S1 Fig) showed that all the lineages are of Pleistocenic origin, since the split between the two main clades A and B occurred around 1.87 mya (1.69 ± 0.2). Within clade A, the supported node splitting the sub-clade A4 vs. all the others is dated 534000 ya (± 90000) and within clade B the main split (B3 vs B1+B2) dated back to 427000 ya (± 86000). The differentiation of tip haplotypes (when resolved) occurred mainly within the Last Glacial Maximum period (LGM) between 47000 and 22000 years BP before the onset of deglaciation around 21000 years ago [63].

### Allozyme data analysis

Eight loci of the 33 studied resulted as monomorphic (*Nadh-dh*, *Np*, *Sod-1*, *Sod-2*, *Adk*, *Lap*, *Fum*, *Mpi*) while the remaining 25 had from two to four alleles per population (S2 Table). Twenty private or rare alleles were found, 17 of which were from populations from the Central-Western part of the range (CW, samples 15–30). The same CW populations showed higher levels of genetic variation than the Eastern samples (E, samples 1–14). The values of the observed heterozygosity ranged from 0.021 to 0.112 in the CW samples and between 0.007 and 0.068 in the E ones, with the respective mean values of 0.073 vs. 0.020 significantly differing (p = 0.0001). Similarly the allelic richness was between 1.121 and 1.112 in CW samples, while the E samples provided lower values between 1.011 and 1.054, with the respective mean values of 1.025 vs. 1.076 significantly differing (p = 0.0001) (Table 4). Sampled populations were all in Hardy-Weinberg equilibrium except three that showed a heterozygote deficiency at locus *Pep-D*. Linkage disequilibrium was calculated for pairwise loci across all populations and no loci pairs showed significant linkage disequilibrium after Bonferroni correction (p < 9.5 x 10^5^).

**Table 4:** Genetic variability parameters calculated over the 33 allozyme loci scored in *H*. *strinatii*.

Population-Code	N_a10_	N_E_	A_R_	H_o_	H_e_	P_99_
**1-SBR**	**1.182**	**1.020**	**1.017**	**0.013**	**0.017**	**0.152**
				*0*.*038*	*0*.*050*	
**2-SBC**	**1.121**	**1.087**	**1.054**	**0.068**	**0.054**	**0.121**
				*0*.*214*	*0*.*155*	
**3-SSM**	**1.061**	**1.032**	**1.023**	**0.027**	**0.022**	**0.061**
				*0*.*110*	*0*.*091*	
**4-SCG**	**1.121**	**1.044**	**1.030**	**0.022**	**0.030**	**0.091**
				*0*.*074*	*0*.*113*	
**5-SCA**	**1.091**	**1.013**	**1.011**	**0.012**	**0.011**	**0.091**
				*0*.*049*	*0*.*044*	
**6-STS**	**1.091**	**1.036**	**1.022**	**0.007**	**0.022**	**0.091**
				*0*.*032*	*0*.*092*	
**7-SCR**	**1.061**	**1.033**	**1.022**	**0.018**	**0.022**	**0.061**
				*0*.*071*	*0*.*092*	
**8-SBN**	**1.121**	**1.037**	**1.026**	**0.032**	**0.026**	**0.121**
				*0*.*111*	*0*.*085*	
**9-SCO**	**1.091**	**1.036**	**1.024**	**0.015**	**0.024**	**0.091**
				*0*.*066*	*0*.*090*	
**10-SBD**	**1.030**	**1.015**	**1.011**	**0.013**	**0.011**	**0.030**
				*0*.*075*	*0*.*063*	
**11-SAL**	**1.061**	**1.034**	**1.039**	**0.022**	**0.024**	**0.061**
				*0*.*089*	*0*.*085*	
**12-SRA**	**1.121**	**1.070**	**1.020**	**0.024**	**0.038**	**0.091**
				*0*.*088*	*0*.*133*	
**13-SBA**	**1.061**	**1.033**	**1.023**	**0.025**	**0.020**	**0.061**
				*0*.*113*	*0*.*089*	
**14-SIS**	**1.091**	**1.044**	**1.028**	**0.017**	**0.028**	**0.091**
				*0*.*081*	*0*.*101*	
**15-SOR**	**1.152**	**1.071**	**1.053**	**0.053**	**0.049**	**0.151**
				*0*.*131*	*0*.*142*	
**16-SRI**	**1.250**	**1.133**	**1.084**	**0.086**	**0.081**	**0.219**
				*0*.*178*	*0*.*176*	
**17-STA**	**1.182**	**1.079**	**1.048**	**0.049**	**0.051**	**0.151**
				*0*.*130*	*0*.*148*	
**18-SGA**	**1.182**	**1.097**	**1.059**	**0.059**	**0.055**	**0.151**
				*0*.*157*	*0*.*144*	
**19-STO**	**1.455**	**1.156**	**1.095**	**0.093**	**0.083**	**0.394**
				*0*.*169*	*0*.*157*	
**20-SSL**	**1.156**	**1.075**	**1.061**	**0.048**	**0.045**	**0.156**
				*0*.*132*	*0*.*126*	
**21-STR**	**1.062**	**1.031**	**1.021**	**0.021**	**0.020**	**0.061**
				*0*.*083*	*0*.*083*	
**23-SSG**	**1.364**	**1.194**	**1.112**	**0.112**	**0.079**	**0.333**
				*0*.*191*	*0*.*135*	
**24-STC**	**1.333**	**1.180**	**1.105**	**0.105**	**0.104**	**0.272**
				*0*.*191*	*0*.*193*	
**25-STE**	**1.394**	**1.162**	**1.092**	**0.092**	**0.089**	**0.242**
				*0*.*181*	*0*.*176*	
**26-STN**	**1.242**	**1.125**	**1.083**	**0.083**	**0.094**	**0.242**
				*0*.*168*	*0*.*190*	
**27-SLU**	**1.188**	**1.126**	**1.079**	**0.079**	**0.083**	**0.182**
				*0*.*181*	*0*.*194*	
**28-SPE**	**1.125**	**1.090**	**1.084**	**0.083**	**0.109**	**0.151**
				*0*.*225*	*0*.*288*	
**29-SMB**	**1.125**	**1.074**	**1.078**	**0.077**	**0.075**	**0.151**
				*0*.*212*	*0*.*217*	
**30-SAS**	**1.273**	**1.157**	**1.093**	**0.093**	**0.070**	**0.212**
* *	* *	* *		*0*.*190*	*0*.*156*	* *

For each sample are reported: the mean (N_a_) and effective (N_E_) number of alleles; the allelic richness (A_R_); the observed (H_o_) and expected heterozygosity (H_e_); the percentage of polymorphic loci according to the 99% criterion (P_99_).

**Fig 4:**
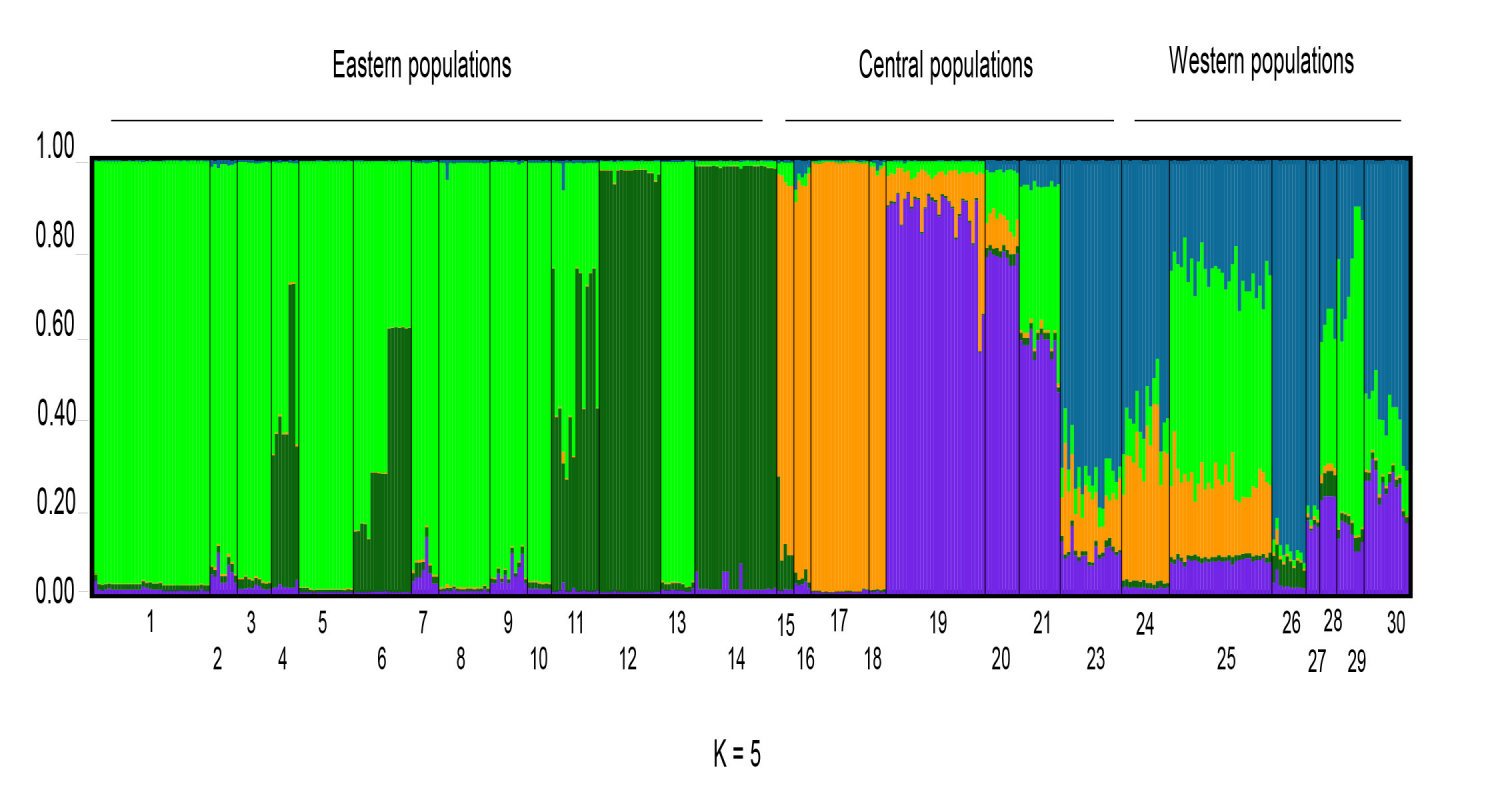
Assignment of *H*. *strinatii* individuals based on allozyme data. Assignment test of specimens (one per column) to the K = 5 clusters identified by the software STRUCTURE based on the 33 scored allozyme loci. Specimens with admixed genomes are identified by columns showing different colours proportional to the percentage of the genome assigned to each of the 5 clusters.

Assignment tests using the Bayesian model-based analysis implemented in STRUCTURE subdivided the studied samples into five clusters (K = 5) as indicated by both the highest value of ΔK (S2 Fig) and by the plateau of the estimated Ln probability. The analysis carried out with BAPS, despite finding the best value for K = 13, reached the plateau for the associated log(ml) between K = 5 and K = 6. The five clusters recovered were geographically consistent and largely in agreement with the groups identified by haplotype analyses (Fig 4). The eastern samples were grouped in two clusters: one comprising samples from 1 to 11 (corresponding to mtA1), and another including samples 12–14 from the “Rapallo area” corresponding to mtA2. The samples 4, 6 and 11 (SCG, STS, SAL) showed some degree of admixture between these two groups. The samples from the “Finalese” area (15–18) were assigned to a cluster corresponding to mtA3, with nearly no admixture, while those from the “Roburent” area (19–21) formed a fourth cluster corresponding to mtA4. The clusters from the westernmost part of the range merged together, including the samples from “San Bartolomeo Hills”, Tenarda and the Maritime French Alps (corresponding to mitochondrial sub-clusters mtB1, mtB2 and mtB3, respectively). Various degrees of admixture were shown and, in particular, all the individuals from population 25 (STE) were strongly admixed with clusters A1 and A3.

**Fig 5:**
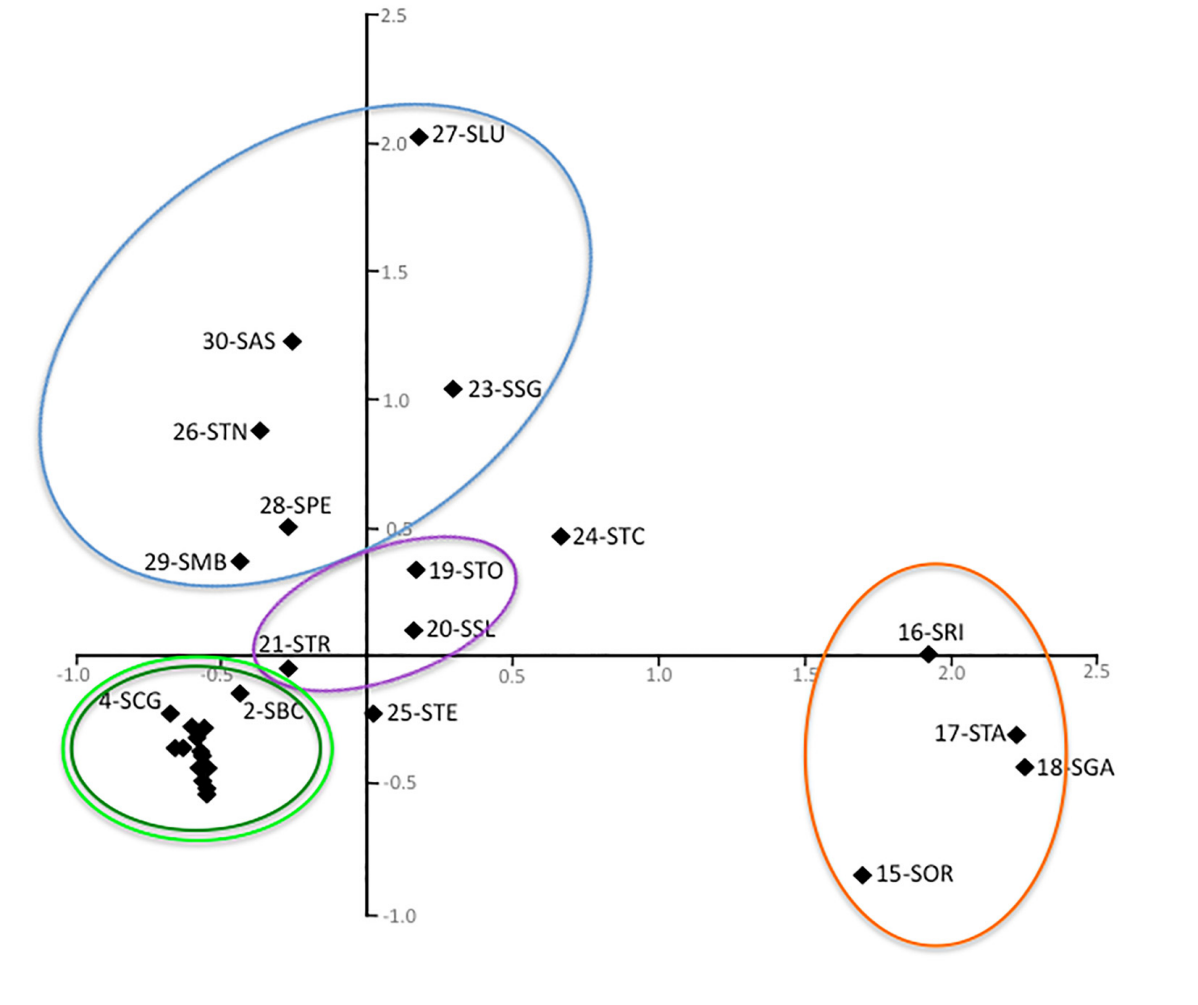
Principal Component Analysis of allozyme allele frequencies in *H*. *strinatii*. Population groups are identified using the same colours as in Fig 1, except for samples 24 (STC) and 25 (STE) which has been left unmarked.

A Principal Component Analysis (PCA) explained 61.0% of variation with two axes, with the first axis explaining 40.6% of variation (p = 0.009). PCA recovered the larger part of the clusters identified by STRUCTURE (Fig 5), the only exceptions being the easternmost samples (clusters mtA1 + mtA2), which were lumped together. The samples from the Finalese area (15–18, cluster mtA3), which showed no admixed genotypes, were clearly separated, while the samples 24 and 25 (STC, STE) showed an intermediate position in accordance with the high level of admixture shown by the assignment analysis.

The AMOVA carried out took into consideration the five clusters inferred from STRUCTURE analysis (Table 2) and showed that the within populations and the between clusters levels mostly contributed to the total variation (47% and 38%, respectively, p < 0.0001 for both), while a smaller but significant fraction of total variation was recovered among populations within the groups (15%, p < 0.0001).

The pairwise F_st_ estimates were always highly significant except when comparing samples from the eastern part of the range (1–11) where only 21 comparisons out of 55 were significant (S2 Table). The overall F_st_ was 0.54 and the highest values (above 0.70) were recorded when comparing the samples from the “Finalese” (15–18) vs. all the others with particularly high values vs. the eastern samples (even above 0.80). To investigate the correspondence of population genetic structuring to a model of IBD, F_st_ and geographic distance values were compared by a Mantel test. The results obtained showed a positive correlation across the whole range of the species (r = 0.192, p = 0.0009) as well as within the eastern part of the range (samples 1–14, r = 0.351, p = 0.0080), while IBD did not hold for the central-western populations.

## Discussion

### Evolutionary process and diversification in *H*. *strinatii*

The results obtained, irrespective of the marker used, showed substantial geographical structuring in *H*. *strinatii* despite the small range size. Large genetic divergence over small geographic scales is quite common in plethodontids, especially if living in geographically old areas [64]. However, the geographic pattern of diversity shown in *H*. *strinatii* is not homogeneous across its range suggesting different processes leading to the extant Eastern (E, 1–14), and Central-Western (CW, 15–30) population groups. Different levels of genetic variation in the E vs. CW populations were concordantly revealed by both mt and nc markers with E populations characterized by a low level of genetic diversity. A single main haplotype is shared by the larger part of E samples within each of the two sub-clades living in this area (A1, A2) together with a few rare singleton differentiated haplotypes. Also, the lowest values for allozyme variability parameters are recorded from these populations irrespective of the sample size. The pattern of differentiation between E populations conforms to the IBD model, linking genetic and geographic distances along a NW-SE axis. Since small population size and low gene flow, which are usually invoked to explain low genetic variation [65], are general features of *Hydromantes*, they cannot account for the lower genetic variation of the E *H*. *strinatii* populations. Our data rather support the idea that this pattern is due to a recent expansion in the E part of the range: significance of neutrality tests and mismatch analysis, low genetic variability, the presence of a few, widespread haplotypes and the geographic pattern of genetic diversification following an “expansion route”, are all in agreement with a recent expansion event [66–68].

In the CW part of the range our data highlighted a higher genetic fragmentation. Nearly each population has its own haplotype, and all the F_st_ values for pairwise comparisons are highly significant. The IBD model does not hold in this area: there is no relationship between genetic and geographic distance with highly differentiated sample pairs found in the range of a few kilometres. Both nc and mt markers identified 3 groups in the C part of the range, but in the W area, the two data sets showed some incongruence. The samples from the “Finalese” (15–18) formed a monophyletic group with the E samples according to mt data, while the same populations resulted as the most differentiated according to the allozymes (Fig 5). In the W part of the range (samples 22–30) the allozymes identified one sub-clade vs. the two recovered by mt data with the allozyme cluster characterized by high admixture levels (Fig 4). This incongruence between mt and nc data can be explained by secondary contacts between differentiated lineages possibly coupled with sex biased dispersal, which is a hypothesis that has been frequently used to reconcile contrasting patterns of nc and mt markers in plethodontids. Allopatric divergence is considered a rule in salamanders, since their low vagility promotes genetic fragmentation as soon as a species disperses over a relatively wide area, especially if this is characterized by complex orography and paleogeographic history [64]. Higher mutation rates and smaller population sizes of mt vs. nc genes would account for the deeper differentiation achieved by mt, while the frequent phylopatry of female salamanders with respect to males would make nc genes merging faster than mt ones [69]. Thus, in many plethodontids allozymes are supposed to reflect recent and ongoing interactions between taxa and populations (i.e., are able to highlight contact zones and gene flow), while mtDNA bears the signature of ancient, deep events [64,70]. Recent data show a broad correspondence between the presence of “misplaced” haplotypes into a nuclear gene pool with introgression events and interpreting the cito-nuclear discordance as support for the mito-genome introgression hypothesis [71–73]. Accordingly, experimental data showed that in *Hydromantes* species introgression may lead to the capture of “foreign” haplotypes into the gene pool of one species although maintaining differentiated nuclear genomes [74]. In this view, the nesting of the “Finalese” group into the mitochondrial subclade A opposed to its external position according to nuclear data can be explained by a contact between the two sub-clades followed by mtDNA introgression into the “Finalese” populations. Secondary contacts may also be invoked to explain the admixture occurring throughout the westernmost neighbouring sub-clades, where the clear diversification of mt lineages B1, B2 and B3 is coupled with nuclear admixture in the same samples (22–30) and where the highly divergent “Finalese” genome components (orange bars in Fig 4) are found in the admixed and geographically close samples 20 and 19, this latter located less than 10 kilometres far from the nearest sample from the “Finalese” group (pop. n. 16, mtA3) and on the same karstic formation (named “Dolomie di San Pietro ai Monti”). However, part of the admixture observed in these samples (22–30) was due to E alleles (green bars in populations 20–21, 25, 28–29; Fig 4). In this case secondary contacts are unlikely, since they would postulate exchanges between geographically distant groups, so that the retention of ancestral polymorphism seems to be a more likely hypothesis. Accordingly, the signature observed in the westernmost part of the range is that typical of incomplete lineage sorting: high genetic variation is coupled with the coexistence of differentiated clades in the same area as a result of the coalescent process making alleles present in the common ancestor still retained in descendent lineages. Since it is a stochastic process, shared alleles are expected to be randomly distributed in the descendant populations instead of being concentrated along the boundaries between the lineages, as would be the case if secondary contacts would occur, and is in agreement with the pattern reported for *H*. *strinatii* [75,76].

### The combined effects of paleoclimatic conditions and localized refugia in *H*. *strinatii*

The data presented showed high levels of genetic heterogeneity in *H*. *strinatii*: most localities had their own haplotypes and even allozymes were able to show highly differentiated populations over a few kilometres. This is in agreement with both literature data, since a strong geographic structure is typical of *Hydromantes*, and the biological and ecological features of the species characterized by low dispersal and high fragmentation [14,15,17,77]. However, the very same features are apparently in contrast with the high levels of genetic diversity recorded. Long evolutionary history and large, stable population size are frequently identified as the mechanisms generating and maintaining high genetic diversity, and are also recognized in plethodontid salamanders as for example *Batrachoseps major* [78]. This is not the case for *H*. *strinatii*, which has very small populations with a census size usually in the order of tens [10,16] and a relatively recent history of diversification when compared to other plethodontids showing similar genetic patterns. Here the split between the clades A and B goes back about 1.7 million years ago and the diversification within each of the two is dating between 427 ka and 534 ka years ago, while much longer divergence times characterize the main clades of other plethodontid species [18,78]. The co-occurrence of different evolutionary processes as secondary contacts and incomplete lineage sorting may help explaining the high levels of genetic diversity recorded in *H*. *strinatii* but other factors are likely to contribute too. Populations of *Hydromantes* may persist for a long time showing a stable demography despite their small size thanks to their association with subterranean retreats. The importance of localized refugia in shaping the genetic structure of species has recently been stressed in a variety of organisms from plethodontids [79] to invertebrates [80–82] and plants [3,4,83]. Within this context, the uniqueness of troglophiles species starts emerging. Since they are able to live in both epigean and subterranean habitats, they can manage to maintain their small populations stable and safe in the face of paleoclimatic changes so retaining their own genetic diversity. High haplotype and nucleotide diversity is the signature of historically stable populations and has been recently interpreted as the inheritance of localized refugia when associated with high geographic structuring and a lack of population expansion [2,84]. These expectations are satisfied by our data from the CW sub-clades of *H*. *strinatii*, which showed strong genetic/geographic structuring, high haplotipic and nucleotidic diversity and no signs of recent expansion. This witnesses a prolonged persistence of these populations in their cave retreats but further considerations on the peculiar paleoclimatic history of the area may add to the general picture. The Ligurian and Maritime Alps are well known glacial refugia since these areas remained at the borders of the ice sheets during Pleistocene glaciations [21,85–89]. Recent studies have shown that these kind of glacial refugia in both the Roya Valley (Maritime French Alps) and in the “Finalese” are characterized by high levels of species richness and endemism occurrence and by high genetic variation of local populations as was observed in *H*. *strinatii* [19,21,86]. The bulk of data produced in the last years added a new scenario to the classical one, which interpreted the high genetic diversity of populations from the Maritime and Ligurian Alps as due to secondary contacts between the Italian peninsular and European lineages [62,90,91]. According to recent data, the Maritime and Ligurian Alps are interpreted as a further glacial refugium and are thus characterized by a high genetic variation of local populations, which persisted here even during the Last Glacial Maximum and then gave birth to hybridization between locally originated lineages as witnessed by *H*. *strinatii* too [22,24,92].

## Conclusions

The study of mt and nc markers in *H*. *strinatii* provided a complex picture, suggesting that the action of different evolutionary processes could be identified despite the low population size typical of this species enhancing the effects of the genetic drift and thus introducing some randomness in the general pattern of variation.

In the CW part of the range, the observed phylogeographic pattern is in agreement with the persistence of local populations due both to the paleoclimatic history of the Ligurian and Maritime Alps, which remained sheltered from ice cover during the Pleistocene glacial episodes, and to the troglophyle habits of the species that is able to use subterranean retreats when climatic conditions become too harsh. The maintenance of high genetic variation is due to the co-occurrence of different mechanisms. These include isolation, promoting divergence and therefore producing clearcut differences in allele frequencies as is the case in the highly isolated “Finalese” area, but also nuclear admixture and mt introgression through secondary contact between geographically nearby lineages. Therefore, in these populations the retention of polymorphism through small populations stability seems to be a relevant mechanism explaining their high genetic variation. This finding strengthens the idea that persistence in small, localized refugia may act efficaciously on tightly associated species in maintaining unusually high levels of genetic diversity through the retention of genetic variation at the intrapopulation level and through the maintenance of any new genotype acquired in the localized refugia.

## Supporting Information

S1 Fig Mitochondrial chronogram for *H*. *strinatii*. Maximum clade credibility tree obtained from the mitochondrial dataset. Red bars at nodes represent the 95% Highest Posterior Densities of node ages. The split node between *H*. *strinatii* and Eastern Sardinian species (*H*. *supramontis* and *H*. *imperialis*) was constrained at 5.33 ma, which is the end of the Messinian salinity crisis.
(TIF)

S2 Fig Results from the ∆K analysis. Methods following Evanno et al. [59], as implemented in Structure Harvester [60]
(TIF)

S1 Table List of allozyme loci scored on *H*. *strinatii*. (DOC)

S2 Table Allozyme allele frequencies in *H*. *strinatii*. Allozyme allele frequencies per population at the 25 polymorphic loci out of the 33 scored.
(DOCX)

S3 Table Pairwise F_st_ values. Pairwise fixation index (F_st_) values based on allozyme data. * p <0.05; ** p < 0.01; *** p < 0.001;—not significant
(PDF)

S1 File Optimal clustering in *H*. *strinatii*. Plot of the number of groups (K) obtained: (a) by plotting F_ct_ vs. K values according to SAMOVA (mt data); (b) by plotting log marginal likelihood vs. K values according to BAPS (b_1_, mt data; b_2_, nuclear data).
(XLS)
