# Supplementary figures and images for: Persistence, Isolation and Diversification of a Naturally Fragmented Species in Local Refugia: The Case of Hydromantes strinatii
Source: PLoS One. 2015 Jun 24;10(6):e0131298. doi: 10.1371/journal.pone.0131298 (PMC4479377; doi:10.1371/journal.pone.0131298)

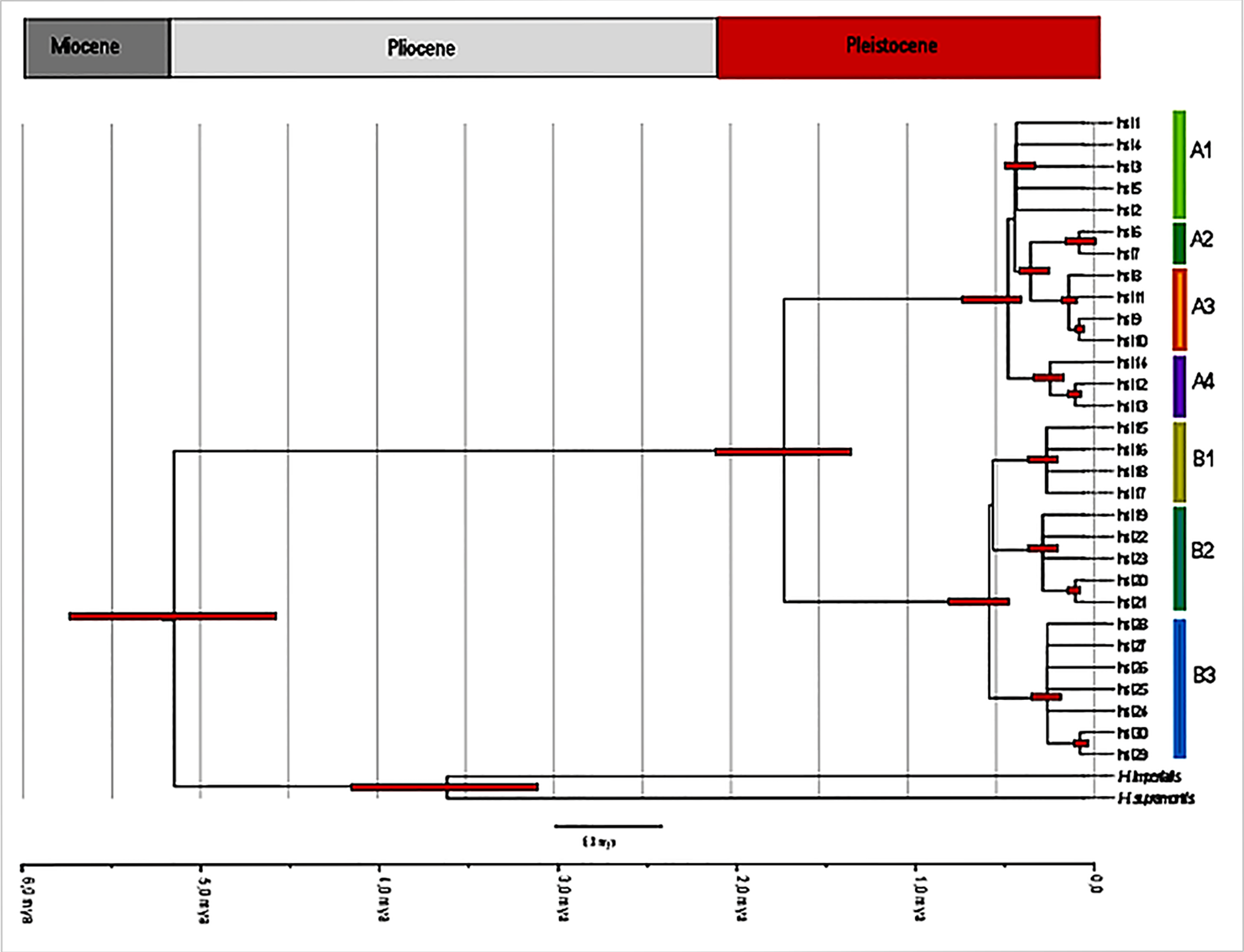

Supplement: S1 Fig — Maximum clade credibility tree obtained from the mitochondrial dataset. Red bars at nodes represent the 95% Highest Posterior Densities of node ages. The split node between H. strinatii and Eastern Sardinian species (H. supramontis and H. imperialis) was constrained at 5.33 ma, which is the end of the Messinian salinity crisis. (TIF) [file pone.0131298.s001.tif]

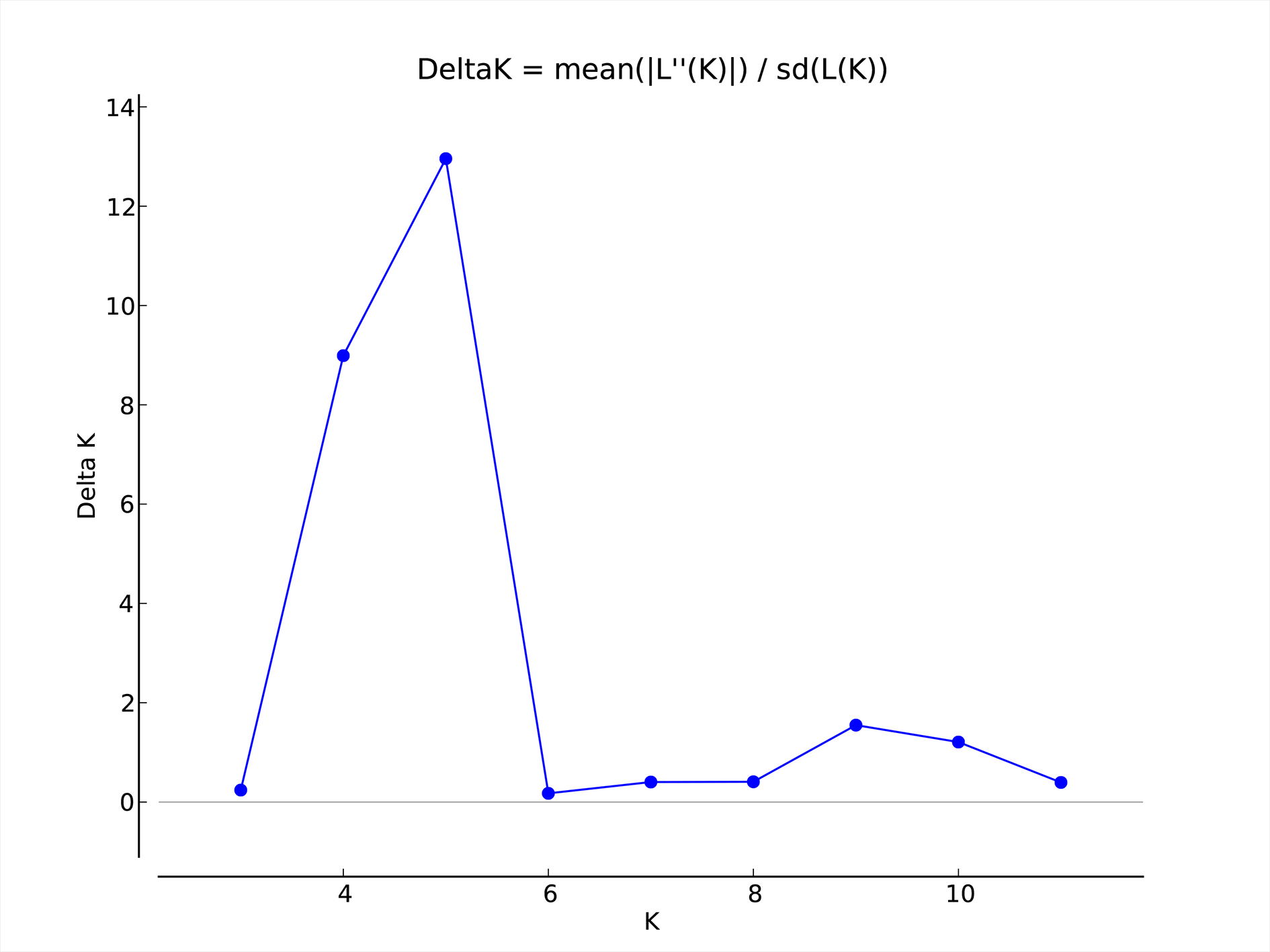

Supplement: S2 Fig — Methods following Evanno et al. [59], as implemented in Structure Harvester [60] (TIF) [file pone.0131298.s002.tif]
