# Supplementary material for: Persistence, Isolation and Diversification of a Naturally Fragmented Species in Local Refugia: The Case of Hydromantes strinatii
Source: PLoS One. 2015 Jun 24;10(6):e0131298. doi: 10.1371/journal.pone.0131298 (PMC4479377; doi:10.1371/journal.pone.0131298)
Supplement: S1 Table — (DOC) [file pone.0131298.s003.doc]

| **Table S1. List of allozyme loci scored on *H. strinatii*.** | | |
| --- | --- | --- |
| **Enzyme** | **E.C. number** | **Encoding loci** |
| Lactate dehydrogenase | EC 1.1.1.27 | ***Ldh-1*** |
|  |  | ***Ldh-2*** |
| Malate dehydrogenase | EC 1.1.1.37 | ***Mdh-1*** |
|  |  | ***Mdh-2*** |
| Malate dehydr. NADP+-dependent | EC 1.1.1.40 | ***Mdhp-1*** |
|  |  | ***Mdhp-2*** |
| Glucose-6-phosphate dehydrogenase | EC 1.1.1.49 | ***G6pdh*** |
| Glyceraldehyde 3-phosphate dehydr. | EC1.2.1.12 | ***Gapdh*** |
| Superoxide dismutase | EC 1.15.1.1 | ***Sod-1*** |
|  |  | ***Sod-2*** |
| Nucleoside phosphorilase | EC 2.4.2.1 | ***Np*** |
| Aspartate aminotransferase | EC 2.6.1.1 | ***Aat-1*** |
|  |  | ***Aat-2*** |
| Creatine kinase | EC 2.7.3.2 | ***Ck*** |
| Adenosine kinase | EC 2.7.1.20 | ***Adk*** |
| Peptidase C | EC 3.4.11.1 | ***PepC-2*** |
| Mannose phosphate isomerase | EC 5.3.1.8 | ***Mpi*** |
| Glucose phosphate isomerase | EC 5.3.1.9 | ***Gpi*** |
| α- Glycerophosphate dehydrogenase | EC 1.1.1.8 | ***a-GPDH*** |
| 3-Hydroxybutyrate dehydrogenase | EC 1.1.1.30 | ***Hbdh*** |
| Isocitrate dehydrogenase | EC 1.1.1.42 | ***Icdh-1*** |
|  |  | ***Icdh-2*** |
| NADH dehydrogenase | EC 1.6.99.3 | ***NADH-dh*** |
| Peptidase-D | EC 3.4.11.1 | ***Pep-D*** |
| Adenosine deaminase | EC 3.5.4.4 | ***Ada-1*** |
|  |  | ***Ada-2*** |
| Esterase | EC 3.1.1.1 | ***Est*** |
| Leucine aminopeptidase | EC 3.4.11.1 | ***Lap*** |
| Carbonic anhydrase | EC 4.2.1.1 | ***Ca-2*** |
|  |  | ***Ca-3*** |
| Phosphoglucomutase | EC 5.4.2.2 | ***Pgm-1*** |
|  |  | ***Pgm-2*** |
| Fumarase | EC 4.2.1.2 | ***Fum*** |
