# Supplementary material for: Persistence, Isolation and Diversification of a Naturally Fragmented Species in Local Refugia: The Case of Hydromantes strinatii
Source: PLoS One. 2015 Jun 24;10(6):e0131298. doi: 10.1371/journal.pone.0131298 (PMC4479377; doi:10.1371/journal.pone.0131298)
Supplement: S2 Table — Allozyme allele frequencies per population at the 25 polymorphic loci out of the 33 scored. (DOCX) [file pone.0131298.s004.docx]

**Table S2.**

**Allozyme allele frequencies.** Allozyme allele frequencies per population at the 25 polymorphic loci out of the 33 scored

|  | **Sample** | | | | | | | | | | | | | | |
| --- | --- | --- | --- | --- | --- | --- | --- | --- | --- | --- | --- | --- | --- | --- | --- |
| **Locus** | SBR | SBC | SSM | SCG | SCA | STS | SCR | SMV | SCO | SBD | SAL | SRA | SBA | SIS | SOR |
| **Allele** | 1 | 2 | 3 | 4 | 5 | 6 | 7 | 8 | 9 | 10 | 11 | 12 | 13 | 14 | 15 |
| ***a-Gpdh*** |  |  |  |  |  |  |  |  |  |  |  |  |  |  |  |
| *85* | --- | --- | --- | --- | --- | --- | --- | --- | --- | --- | --- | --- | --- | --- | --- |
| *90* | --- | --- | --- | --- | --- | --- | --- | --- | --- | --- | --- | --- | --- | --- | 1.000 |
| *95* | 1.000 | 0.667 | 1.000 | 1.000 | 1.000 | 1.000 | 1.000 | 1.000 | 0.938 | 1.000 | 1.000 | 1.000 | 1.000 | 1.000 | --- |
| *100* | --- | 0.333 | --- | --- | --- | --- | --- | --- | 0.062 | --- | --- | --- | --- | --- | --- |
| *108* | --- | --- | --- | --- | --- | --- | --- | --- | --- | --- | --- | --- | --- | --- | --- |
| Ldh-1 |  |  |  |  |  |  |  |  |  |  |  |  |  |  |  |
| *97* | 1.000 | 1.000 | 1.000 | 1.000 | 1.000 | 1.000 | 1.000 | 0.938 | 1.000 | 1.000 | 1.000 | 1.000 | 1.000 | 1.000 | 1.000 |
| *107* | --- | --- | --- | --- | --- | --- | --- | 0.062 | --- | --- | --- | --- | --- | --- | --- |
| Ldh-2 |  |  |  |  |  |  |  |  |  |  |  |  |  |  |  |
| *108* | 1.000 | 0.875 | 1.000 | 1.000 | 1.000 | 1.000 | 1.000 | 1.000 | 1.000 | 1.000 | 1.000 | 1.000 | 1.000 | 1.000 | 1.000 |
| *112* | --- | 0.125 | --- | --- | --- | --- | --- | --- | --- | --- | --- | --- | --- | --- | --- |
| Hbdh |  |  |  |  |  |  |  |  |  |  |  |  |  |  |  |
| *105* | 1.000 | 1.000 | 1.000 | 1.000 | 1.000 | 1.000 | 1.000 | 1.000 | 1.000 | 1.000 | 1.000 | 1.000 | 1.000 | 1.000 | 1.000 |
| *120* | --- | --- | --- | --- | --- | --- | --- | --- | --- | --- | --- | --- | --- | --- | --- |
| Mdh-1 |  |  |  |  |  |  |  |  |  |  |  |  |  |  |  |
| *90* | --- | --- | --- | --- | --- | --- | --- | --- | --- | --- | --- | --- | --- | --- | --- |
| *100* | 1.000 | 1.000 | 1.000 | 1.000 | 1.000 | 1.000 | 1.000 | 1.000 | 1.000 | 1.000 | 1.000 | 1.000 | 1.000 | 1.000 | 1.000 |
| Mdh-2 |  |  |  |  |  |  |  |  |  |  |  |  |  |  |  |
| *107* | --- | --- | --- | --- | --- | --- | --- | --- | --- | --- | --- | --- | --- | --- | --- |
| *115* | 1.000 | 1.000 | 1.000 | 1.000 | 0.969 | 0.971 | 1.000 | 0.900 | 1.000 | 1.000 | 1.000 | 1.000 | 1.000 | 1.000 | 1.000 |
| *125* | --- | --- | --- | --- | 0.031 | 0.029 | --- | 0.100 | --- | --- | --- | --- | --- | --- | --- |
| Mdhp-1 |  |  |  |  |  |  |  |  |  |  |  |  |  |  |  |
| *110* | 1.000 | 1.000 | 1.000 | 1.000 | 1.000 | 1.000 | 1.000 | 1.000 | 1.000 | 1.000 | 1.000 | 1.000 | 1.000 | 1.000 | 1.000 |
| *118* | --- | --- | --- | --- | --- | --- | --- | --- | --- | --- | --- | --- | --- | --- | --- |
| *126* | --- | --- | --- | --- | --- | --- | --- | --- | --- | --- | --- | --- | --- | --- | --- |
| ***Mdhp-2*** |  |  |  |  |  |  |  |  |  |  |  |  |  |  |  |
| *94* | 1.000 | 1.000 | 1.000 | 1.000 | 1.000 | 1.000 | 1.000 | 1.000 | 1.000 | 1.000 | 1.000 | 0.900 | 1.000 | 1.000 | 1.000 |
| *103* | --- | --- | --- | --- | --- | --- | --- | --- | --- | --- | --- | 0.100 | --- | --- | --- |
| ***Idh-1*** |  |  |  |  |  |  |  |  |  |  |  |  |  |  |  |
| *90* | --- | --- | --- | --- | --- | --- | --- | --- | --- | --- | --- | --- | --- | --- | --- |
| *94* | 0.983 | 1.000 | 1.000 | 1.000 | 1.000 | 1.000 | 1.000 | 1.000 | 1.000 | 1.000 | 1.000 | 1.000 | 1.000 | 1.000 | 1.000 |
| *103* | 0.017 | --- | --- | --- | --- | --- | --- | --- | --- | --- | --- | --- | --- | --- | --- |
| ***Idh-2*** |  |  |  |  |  |  |  |  |  |  |  |  |  |  |  |
| *97* | --- | --- | --- | --- | --- | --- | --- | --- | --- | --- | --- | --- | --- | --- | --- |
| *100* | 1.000 | 1.000 | 1.000 | 1.000 | 1.000 | 1.000 | 1.000 | 1.000 | 1.000 | 1.000 | 1.000 | 1.000 | 1.000 | 1.000 | 1.000 |
| *110* | --- | --- | --- | --- | --- | --- | --- | --- | --- | --- | --- | --- | --- | --- | --- |
| ***6Pgdh*** |  |  |  |  |  |  |  |  |  |  |  |  |  |  |  |
| *87* | --- | --- | --- | --- | --- | --- | --- | --- | --- | --- | --- | --- | --- | --- | --- |
| *95* | --- | --- | --- | --- | --- | --- | --- | --- | --- | --- | --- | --- | --- | --- | --- |
| *97* | 0.939 | 0.500 | 0.750 | 0.900 | 0.867 | 0.912 | 0.875 | 0.700 | 0.909 | 0.786 | 1.000 | 0.900 | 0.700 | 0.947 | 1.000 |
| *103* | 0.061 | 0.500 | 0.250 | 0.100 | 0.133 | 0.088 | 0.125 | 0.300 | 0.091 | 0.214 | --- | 0.100 | 0.300 | 0.053 | --- |
| ***Gapdh*** |  |  |  |  |  |  |  |  |  |  |  |  |  |  |  |
| *93* | 0.981 | 0.875 | 1.000 | 1.000 | 1.000 | 1.000 | 1.000 | 1.000 | 1.000 | 1.000 | 1.000 | 1.000 | 1.000 | 1.000 | 1.000 |
| *96* | 0.019 | 0.825 | --- | --- | --- | --- | --- | --- | --- | --- | --- | --- | --- | --- | --- |
| *105* | --- | --- | --- | --- | --- | --- | --- | --- | --- | --- | --- | --- | --- | --- | --- |
| ***Aat-1*** |  |  |  |  |  |  |  |  |  |  |  |  |  |  |  |
| *90* | --- | --- | --- | --- | --- | --- | --- | --- | --- | --- | --- | --- | --- | --- | --- |
| *103* | --- | --- | --- | --- | --- | --- | --- | --- | --- | --- | --- | --- | --- | --- | 1.000 |
| *110* | 1.000 | 1.000 | 1.000 | 1.000 | 1.000 | 1.000 | 1.000 | 1.000 | 1.000 | 1.000 | 1.000 | 1.000 | 1.000 | 1.000 | --- |
| *120* | --- | --- | --- | --- | --- | --- | --- | --- | --- | --- | --- | --- | --- | --- | --- |
| ***Aat-2*** |  |  |  |  |  |  |  |  |  |  |  |  |  |  |  |
| *88* | 1.000 | 1.000 | 1.000 | 1.000 | 1.000 | 1.000 | 1.000 | 1.000 | 1.000 | 1.000 | 1.000 | 1.000 | 1.000 | 1.000 | 1.000 |
| *105* | --- | --- | --- | --- | --- | --- | --- | --- | --- | --- | --- | --- | --- | --- | --- |
| ***Ck*** |  |  |  |  |  |  |  |  |  |  |  |  |  |  |  |
| *90* | 1.000 | 1.000 | 1.000 | 1.000 | 1.000 | 1.000 | 1.000 | 1.000 | 1.000 | 1.000 | 1.000 | 1.000 | 1.000 | 1.000 | 1.000 |
| 100 | --- | --- | --- | --- | --- | --- | --- | --- | --- | --- | --- | --- | --- | --- | --- |

*Follows*

*Following*

**Table S2. Allozyme allele frequencies**

|  | **Sample** | | | | | | | | | | | | | | |
| --- | --- | --- | --- | --- | --- | --- | --- | --- | --- | --- | --- | --- | --- | --- | --- |
| **Locus** | SBR | SBC | SSM | SCG | SCA | STS | SCR | SMV | SCO | SBD | SAL | SRA | SBA | SIS | SOR |
| **Allele** | 1 | 2 | 3 | 4 | 5 | 6 | 7 | 8 | 9 | 10 | 11 | 12 | 13 | 14 | 15 |
| ***Pgm-1*** |  |  |  |  |  |  |  |  |  |  |  |  |  |  |  |
| *92* | --- | --- | --- | --- | --- | --- | --- | --- | --- | --- | 0.385 | 0.625 | --- | 0.771 | 0.300 |
| *102* | 0.912 | 1.000 | 1.000 | 0.962 | 1.000 | 1.000 | 1.000 | 1.000 | 1.000 | 1.000 | 0.615 | 0.375 | 1.000 | 0.229 | 0.700 |
| *110* | 0.088 | --- | --- | 0.038 | --- | --- | --- | --- | --- | --- | --- | --- | --- | --- | --- |
| ***Pgm-2*** |  |  |  |  |  |  |  |  |  |  |  |  |  |  |  |
| *86* | --- | --- | --- | --- | --- | --- | --- | --- | --- | --- | --- | --- | --- | --- | --- |
| *92* | 1.000 | 1.000 | 1.000 | 1.000 | 1.000 | 1.000 | 1.000 | 1.000 | 1.000 | 1.000 | 1.000 | 1.000 | 1.000 | 1.000 | 1.000 |
| ***Est-4*** |  |  |  |  |  |  |  |  |  |  |  |  |  |  |  |
| *95* | --- | --- | --- | --- | --- | --- | --- | --- | --- | --- | --- | --- | --- | --- | --- |
| *100* | --- | --- | --- | --- | --- | --- | --- | --- | --- | --- | --- | --- | --- | --- | --- |
| *105* | --- | --- | --- | --- | --- | --- | --- | --- | --- | --- | --- | --- | --- | --- | 0.200 |
| *110* | 1.000 | 1.000 | 1.000 | 1.000 | 1.000 | 1.000 | 1.000 | 1.000 | 1.000 | 1.000 | 1.000 | 1.000 | 1.000 | 1.000 | 0.800 |
| ***Ap*** |  |  |  |  |  |  |  |  |  |  |  |  |  |  |  |
| *95* | --- | --- | --- | --- | --- | --- | --- | --- | --- | --- | --- | --- | --- | --- | 0.200 |
| *100* | 1.000 | 1.000 | 1.000 | 1.000 | 1.000 | 1.000 | 1.000 | 1.000 | 1.000 | 1.000 | 1.000 | 1.000 | 1.000 | 1.000 | 0.800 |
| ***Pep-D*** |  |  |  |  |  |  |  |  |  |  |  |  |  |  |  |
| *100* | --- | --- | --- | --- | --- | --- | --- | --- | --- | --- | --- | --- | --- | --- | --- |
| *110* | 0.958 | 0.800 | 0.800 | 0.833 | 1.000 | 1.000 | 0.667 | 0.933 | 0.615 | 1.000 | 0.875 | 0.833 | 0.833 | 0.833 | 1.000 |
| *115* | 0.042 | 0.200 | 0.200 | 0.167 | --- | --- | 0.333 | 0.067 | 0.385 | --- | 0.125 | 0.167 | 0.167 | 0.167 | --- |
| *125 ß* | --- | --- | --- | --- | --- | --- | --- | --- | --- | --- | --- | --- | --- | --- | --- |
| ***Ada-1*** |  |  |  |  |  |  |  |  |  |  |  |  |  |  |  |
| *85* | 1.000 | 1.000 | 1.000 | 1.000 | 1.000 | 1.000 | 1.000 | 1.000 | 1.000 | 1.000 | 1.000 | 1.000 | 1.000 | 1.000 | 1.000 |
| *98* | --- | --- | --- | --- | --- | --- | --- | --- | --- | --- | --- | --- | --- | --- | --- |
| ***Ada-2*** |  |  |  |  |  |  |  |  |  |  |  |  |  |  |  |
| *100* | --- | --- | --- | --- | --- | --- | --- | --- | --- | --- | --- | --- | --- | --- | --- |
| *103* | --- | --- | --- | --- | --- | --- | --- | --- | --- | --- | --- | --- | --- | --- | 0.900 |
| *106* | --- | --- | --- | --- | --- | --- | --- | --- | --- | --- | --- | --- | --- | --- | --- |
| *110* | 1.000 | 1.000 | 1.000 | 1.000 | 1.000 | 1.000 | 1.000 | 1.000 | 1.000 | 1.000 | 1.000 | 1.000 | 1.000 | 1.000 | 0.100 |
| ***Ca-2*** |  |  |  |  |  |  |  |  |  |  |  |  |  |  |  |
| *90* | 1.000 | 1.000 | 1.000 | 1.000 | 1.000 | 1.000 | 1.000 | 1.000 | 1.000 | 1.000 | 1.000 | 1.000 | 1.000 | 1.000 | 1.000 |
| *100* | --- | --- | --- | --- | --- | --- | --- | --- | --- | --- | --- | --- | --- | --- | --- |
| ***Ca-3*** |  |  |  |  |  |  |  |  |  |  |  |  |  |  |  |
| *82* | --- | --- | --- | --- | --- | --- | --- | --- | --- | --- | --- | --- | --- | --- | --- |
| *90* | 1.000 | 1.000 | 1.000 | 1.000 | 1.000 | 1.000 | 1.000 | 1.000 | 1.000 | 1.000 | 1.000 | 1.000 | 1.000 | 1.000 | 1.000 |
| ***Gpi*** |  |  |  |  |  |  |  |  |  |  |  |  |  |  |  |
| *93* | --- | --- | --- | --- | --- | --- | --- | --- | --- | --- | --- | --- | --- | --- | --- |
| *95* | --- | --- | --- | 0.100 | --- | --- | --- | --- | --- | --- | --- | --- | --- | --- | --- |
| *100* | 1.000 | 1.000 | 1.000 | 0.900 | 1.000 | 1.000 | 1.000 | 1.000 | 1.000 | 1.000 | 1.000 | 1.000 | 1.000 | 1.000 | 1.000 |

*Follows*

*Following*

**Table S2: Allozyme allele frequencies**

|  | **Sample** | | | | | | | | | | | | | |
| --- | --- | --- | --- | --- | --- | --- | --- | --- | --- | --- | --- | --- | --- | --- |
| **Locus** | SRI | STA | SGA | STO | SSL | STR | SSG | STC | STE | STN | SLU | SPE | SMB | SAS |
| **Allele** | 16 | 17 | 18 | 19 | 20 | 21 | 23 | 24 | 25 | 26 | 27 | 28 | 29 | 30 |
| ***a-Gpdh*** |  |  |  |  |  |  |  |  |  |  |  |  |  |  |
| *75* | 0.100 | --- | --- | --- | --- | --- | 0.382 | 0.571 | --- | --- | --- | --- | --- | --- |
| *80* | 0.900 | 1.000 | 1.000 | 0.385 | 0.650 | --- | --- | --- | 0.038 | 0.250 | --- | --- | --- | --- |
| *85* | --- | --- | --- | 0.615 | 0.350 | 1.000 | 0.618 | 0.429 | 0.962 | 0.750 | 0.125 | 1.000 | 0.875 | 1.000 |
| *100* | --- | --- | --- | --- | --- | --- | --- | --- | --- | --- | 0.875 | --- | --- | --- |
| *108* | --- | --- | --- | --- | --- | --- | --- | --- | --- | --- | --- | --- | 0.125 | --- |
| ***Ldh-1*** |  |  |  |  |  |  |  |  |  |  |  |  |  |  |
| *97* | 1.000 | 1.000 | 0.800 | 1.000 | 1.000 | 1.000 | 1.000 | 1.000 | 0.667 | 0.200 | 1.000 | 1.000 | 1.000 | 0.885 |
| *107* | --- | --- | 0.200 | --- | --- | --- | --- | --- | 0.333 | 0.800 | --- | --- | --- | 0.115 |
| ***Ldh-2*** |  |  |  |  |  |  |  |  |  |  |  |  |  |  |
| *108* | 1.000 | 1.000 | 0.900 | 0.955 | 1.000 | 1.000 | 1.000 | 1.000 | 1.000 | 1.000 | 1.000 | 1.000 | 1.000 | 1.000 |
| *112* | --- | --- | 0.100 | 0.045 | --- | --- | --- | --- | --- | --- | --- | --- | --- | --- |
| ***Hbdh*** |  |  |  |  |  |  |  |  |  |  |  |  |  |  |
| *105* | 1.000 | 1.000 | 1.000 | 1.000 | 1.000 | 1.000 | 1.000 | 1.000 | 1.000 | 1.000 | 1.000 | 1.000 | 1.000 | 0.933 |
| *120* | --- | --- | --- | --- | --- | --- | --- | --- | --- | --- | --- | --- | --- | 0.067 |
| ***Mdh-1*** |  |  |  |  |  |  |  |  |  |  |  |  |  |  |
| *90* | --- | --- | --- | 0.020 | --- | --- | 0.038 | 0.077 | 0.024 | 0.050 | --- | --- | --- | --- |
| *100* | 1.000 | 1.000 | 1.000 | 0.980 | 1.000 | 1.000 | 0.962 | 0.923 | 0.9876 | 0.950 | 1.000 | 1.000 | 1.000 | 1.000 |
| ***Mdh-2*** |  |  |  |  |  |  |  |  |  |  |  |  |  |  |
| *107* | --- | --- | --- | --- | --- | --- | --- | --- | 0.240 | 0.250 | --- | --- | --- | --- |
| *115* | 1.000 | 1.000 | 1.000 | 1.000 | 1.000 | 1.000 | 1.000 | 1.000 | 0.760 | 0.750 | 1.000 | 1.000 | 1.000 | 1.000 |
| *125* | --- | --- | --- | --- | --- | --- | --- | --- | --- | --- | --- | --- | --- | --- |
| ***Mdhp-1*** |  |  |  |  |  |  |  |  |  |  |  |  |  |  |
| *110* | 1.000 | 1.000 | 1.000 | 1.000 | 1.000 | 1.000 | 0.250 | 0.107 | 0.967 | 0.750 | 0.350 | 1.000 | 1.000 | 0.654 |
| *118* | --- | --- | --- | --- | --- | --- | 0.750 | 0.893 | 0.033 | 0.250 | 0.650 | --- | -- | 0.308 |
| *126* | --- | --- | --- | --- | --- | --- | --- | --- | --- | --- | --- | --- | --- | 0.038 |
| ***Mdhp-2*** |  |  |  |  |  |  |  |  |  |  |  |  |  |  |
| *94* | 0.300 | 0.900 | 1.000 | 1.000 | 1.000 | 1.000 | 0.806 | 1.000 | 0.717 | 1.000 | 0.500 | 0.300 | 0.300 | 0.692 |
| *103* | 0.700 | 0.100 | --- | --- | --- | --- | 0.194 | --- | 0.283 | --- | 0.500 | 0.700 | 0.700 | 0.308 |
| ***Idh-1*** |  |  |  |  |  |  |  |  |  |  |  |  |  |  |
| *90* | --- | --- | --- | --- | --- | --- | 0.033 | --- | --- | --- | --- | --- | --- | --- |
| *94* | 1.000 | 1.000 | 1.000 | 0.981 | 1.000 | 1.000 | 0.967 | 1.000 | 1.000 | 1.000 | 1.000 | 1.000 | 1.000 | 1.000 |
| *103* | --- | --- | --- | 0.019 | --- | --- | --- | --- | --- | --- | --- | --- | --- | --- |
| ***Idh-2*** |  |  |  |  |  |  |  |  |  |  |  |  |  |  |
| *97* | --- | --- | --- | --- | --- | --- | 0.033 | --- | --- | --- | 0.625 | 0.600 | 0.250 | 0.778 |
| *100* | 1.000 | 1.000 | 1.000 | 1.000 | 0.950 | 1.000 | 1.000 | 1.000 | 1.000 | 1.000 | 0.375 | 0.400 | 0.750 | 0.222 |
| *110* | --- | --- | --- | --- | 0.050 | --- | 0.033 | --- | --- | --- | --- | --- | --- | --- |
| ***6Pgdh*** |  |  |  |  |  |  |  |  |  |  |  |  |  |  |
| *87* | 0.300 | 0.353 | 0.550 | 0.400 | 0.650 | 0.792 | 0.778 | 0.333 | 0.250 | 0.800 | 0.875 | 0.100 | 0.312 | 0.692 |
| *95* | --- | --- | --- | --- | --- | --- | --- | 0.083 | --- | --- | --- | --- | --- | --- |
| *97* | 0.600 | 0.559 | 0.350 | 0.200 | 0.350 | 0.208 | 0.222 | 0.583 | 0.700 | 0.200 | 0.125 | 0.900 | 0.668 | 0.308 |
| *103* | 0.100 | 0.098 | 0.100 | 0.010 | --- | --- | --- | --- | 0.050 | --- | --- | --- | --- | --- |
| ***Gapdh*** |  |  |  |  |  |  |  |  |  |  |  |  |  |  |
| *93* | 1.000 | 1.000 | 1.000 | 0.542 | 1.000 | 1.000 | 0.929 | 0.875 | 1.000 | 1.000 | 1.000 | 1.000 | 1.000 | 1.000 |
| *96* | --- | --- | --- | 0.458 | --- | --- | --- | --- | --- | --- | --- | --- | --- | --- |
| *105* | --- | ..---.. | --- | --- | --- | --- | 0.071 | 0.125 | --- | --- | --- | --- | --- | --- |
| ***Aat-1*** |  |  |  |  |  |  |  |  |  |  |  |  |  |  |
| *90* | --- | --- | --- | 0.034 | --- | --- | --- | --- | 0.037 | --- | --- | --- | --- | --- |
| *103* | 0.800 | 1.000 | 1.000 | 0.121 | --- | --- | 0.111 | 0.423 | 0.400 | --- | --- | --- | --- | --- |
| *110* | 0.200 | --- | --- | 0.845 | 0.950 | 1.000 | 0.889 | 0.577 | 0.567 | 1.000 | 1.000 | 1.000 | 1.000 | 1.000 |
| *120* | --- | --- | --- | --- | 0.050 | --- | --- | --- | --- | --- | --- | --- | --- | --- |
| ***Aat-2*** |  |  |  |  |  |  |  |  |  |  |  |  |  |  |
| *88* | 1.000 | 1.000 | 1.000 | 0.983 | 1.000 | 1.000 | 1.000 | 1.000 | 1.000 | 1.000 | 1.000 | 1.000 | 1.000 | 1.000 |
| *105* | --- | --- | --- | 0.017 | --- | --- | --- | --- | --- | --- | --- | --- | --- | --- |
| ***Ck*** |  |  |  |  |  |  |  |  |  |  |  |  |  |  |
| *90* | 1.000 | 1.000 | 1.000 | 1.000 | 1.000 | 1.000 | 1.000 | 1.000 | 1.000 | 0.400 | 1.000 | 1.000 | 1.000 | 1.000 |
| *100* | --- | --- | --- | --- | --- | --- | --- | --- | --- | 0.600 | -- | --- | --- | --- |

*Following*

**Table S2: Allozyme allele frequencies**

|  | **Sample** | | | | | | | | | | | | | |
| --- | --- | --- | --- | --- | --- | --- | --- | --- | --- | --- | --- | --- | --- | --- |
| **Locus** | SRI | STA | SGA | STO | SSL | STR | SSG | STC | STE | STN | SLU | SPE | SMB | SAS |
| **Allele** | 16 | 17 | 18 | 19 | 20 | 21 | 23 | 24 | 25 | 26 | 27 | 28 | 29 | 30 |
| ***Pgm-1*** |  |  |  |  |  |  |  |  |  |  |  |  |  |  |
| *92* | 0.300 | 0.235 | 0.400 | --- | --- | --- | --- | --- | 0.033 | --- | --- | --- | --- | --- |
| *102* | 0.700 | 0.765 | 0.600 | 1.000 | 1.000 | 1.000 | 1.000 | 1.000 | 0.967 | 1.000 | 1.000 | 1.000 | 1.000 | 1.000 |
| ***Pgm-2*** |  |  |  |  |  |  |  |  |  |  |  |  |  |  |
| *86* | --- | --- | --- | 0.020 | --- | --- | --- | --- | --- | --- | --- | --- | --- | --- |
| *92* | 1.000 | 1.000 | 1.000 | 0.980 | 1.000 | 1.000 | 1.000 | 1.000 | 1.000 | 1.000 | 1.000 | 1.000 | 1.000 | 1.000 |
| ***Est-4*** |  |  |  |  |  |  |  |  |  |  |  |  |  |  |
| *95* | --- | --- | --- | --- | --- | --- | 0.333 | 0.071 | --- | --- | --- | --- | --- | --- |
| *100* | --- | --- | --- | --- | --- | --- | --- | --- | 0.115 | --- | --- | --- | --- | --- |
| *105* | 0.700 | 0.917 | 0.900 | 0.457 | --- | --- | 0.167 | 0.143 | 0.270 | 0.200 | 0.500 | 0.500 | --- | 0.462 |
| *110* | 0.300 | 0.083 | 0.100 | 0.543 | 1.000 | 1.000 | 0.500 | 0.786 | 0.615 | 0.800 | 0.500 | 0.500 | 1.000 | 0.538 |
| ***Ap*** |  |  |  |  |  |  |  |  |  |  |  |  |  |  |
| *95* | --- | --- | --- | --- | --- | --- | --- | --- | --- | --- | --- | --- | --- | --- |
| *100* | 1.000 | 1.000 | 1.000 | 1.000 | 1.000 | 1.000 | 1.000 | 1.000 | 1.000 | 1.000 | 1.000 | 1.000 | 1.000 | 1.000 |
| ***Pep-D*** |  |  |  |  |  |  |  |  |  |  |  |  |  |  |
| *90* | 1.000 | 0.833 | 1.000 | 1.000 | 1.000 | 1.000 | 0.417 | 0.667 | 1.000 | 1.000 | 1.000 | 0.667 | 0.750 | 0.400 |
| *96* | --- | 0.167 | --- | --- | --- | --- | 0.583 | 0.333 | --- | --- | --- | 0.333 | 0.250 | 0.600 |
| ***Ada-1*** |  |  |  |  |  |  |  |  |  |  |  |  |  |  |
| *85* | --- | --- | --- | --- | --- | --- | --- | --- | --- | --- | --- | --- | --- | --- |
| *98* | 1.000 | 1.000 | 1.000 | 1.000 | 1.000 | 1.000 | 1.000 | 1.000 | 1.000 | 1.000 | 1.000 | 1.000 | 1.000 | 0.077 |
| C | --- | --- | --- | --- | --- | --- | --- | --- | --- | --- | 0.923 |  |  |  |
| ***Ada-2*** |  |  |  |  |  |  |  |  |  |  |  |  |  |  |
| *100* | --- | --- | --- | --- | --- | --- | --- | --- | --- | --- | --- | --- | --- | --- |
| *103* | 0.900 | 1.000 | 1.000 | 0.172 | 0.222 | 0.208 | 0.389 | 0.667 | 1.000 | --- | 1.000 | 1.000 | 1.000 | 1.000 |
| *106* | --- | --- | --- | --- | --- | --- | --- | --- | --- | --- | --- | --- | --- | --- |
| *110* | 0.100 | --- | --- | 0.828 | 0.778 | 0.792 | 0.611 | 0.333 | --- | --- | --- | --- | --- | --- |
| ***Ca-2*** |  |  |  |  |  |  |  |  |  |  |  |  |  |  |
| *90* | 1.000 | 1.000 | 1.000 | 0.958 | 1.000 | 1.000 | 1.000 | 1.000 | 1.000 | 1.000 | 1.000 | 1.000 | 1.000 | 1.000 |
| *100* | --- | --- | --- | 0.042 | --- | --- | --- | --- | --- | --- | --- | --- | --- | --- |
| ***Ca-3*** |  |  |  |  |  |  |  |  |  |  |  |  |  |  |
| *82* | --- | --- | --- | --- | --- | --- | --- | --- | --- | --- | --- | --- | 0.038 | --- |
| *90* | 1.000 | 1.000 | 1.000 | 1.000 | 1.000 | 1.000 | 1.000 | 1.000 | 1.000 | 1.000 | 1.000 | 1.000 | 0.962 | 1.000 |
| ***Gpi*** |  |  |  |  |  |  |  |  |  |  |  |  |  |  |
| *93* | --- | --- | --- | --- | --- | --- | --- | --- | --- | --- | 0.050 | --- | --- | --- |
| *95* | --- | --- | --- | --- | --- | --- | --- | --- | --- | --- | --- | --- | --- | --- |
| *100* | 1.000 | 1.000 | 1.000 | 1.000 | 1.000 | 1.000 | 1.000 | 1.000 | 1.000 | 1.000 | 0.950 | 1.000 | 1.000 | 1.000 |
