# Supplementary material for: Persistence, Isolation and Diversification of a Naturally Fragmented Species in Local Refugia: The Case of Hydromantes strinatii
Source: PLoS One. 2015 Jun 24;10(6):e0131298. doi: 10.1371/journal.pone.0131298 (PMC4479377; doi:10.1371/journal.pone.0131298)
Supplement: S3 Table — Pairwise fixation index (Fst) values based on allozyme data. * p <0.05; ** p < 0.01; *** p < 0.001;—not significant (PDF) [file pone.0131298.s005.pdf]

**Pairwise $F_{st}$ values.** Pairwise fixation index ($F_{st}$) values based on allozyme data. * p <0.05; ** p < 0.01; *** p < 0.001; $^{---}$ not significant

| Pop | 1-SBR | 2-SBC | 3-SSM | 4-SCG | 5-SCA | 6-STS | 7-SCR | 8-SBN | 9-SCO | 10-SBD | 11-SAL | 12-SRA | 14-SBA | 13-SIS | 15-SOR | 16-SRI | 17-STA | 18-SGA | 19-STO | 20-SSL | 21-STR | 23-SSG | 24-STC | 25-STE | 26-STN | 27-SLU | 28-SPE | 29-SMB |
|---|---|---|---|---|---|---|---|---|---|---|---|---|---|---|---|---|---|---|---|---|---|---|---|---|---|---|---|---|
| 2 | 0.2793$^{**}$ | | | | | | | | | | | | | | | | | | | | | | | | | | | |
| 3 | 0.0536$^{---}$ | 0.0751$^{---}$ | | | | | | | | | | | | | | | | | | | | | | | | | | |
| 4 | 0.3447$^{*}$ | 0.2704$^{*}$ | 0.2425$^{*}$ | | | | | | | | | | | | | | | | | | | | | | | | | |
| 5 | 0.0074$^{---}$ | 0.2835$^{**}$ | 0.0507$^{---}$ | 0.4417$^{***}$ | | | | | | | | | | | | | | | | | | | | | | | | |
| 6 | 0.2586$^{***}$ | 0.3164$^{***}$ | 0.1905$^{**}$ | -0.0733$^{---}$ | 0.3330$^{***}$ | | | | | | | | | | | | | | | | | | | | | | | |
| 7 | 0.0342$^{---}$ | 0.1177$^{---}$ | -0.0278$^{---}$ | 0.1979$^{---}$ | 0.1299$^{---}$ | 0.1842$^{***}$ | | | | | | | | | | | | | | | | | | | | | | |
| 8 | 0.1027$^{---}$ | 0.1313$^{---}$ | 0.0030$^{---}$ | 0.3184$^{**}$ | 0.0377$^{---}$ | 0.2894$^{***}$ | 0.0862$^{---}$ | | | | | | | | | | | | | | | | | | | | | |
| 9 | 0.0639$^{---}$ | 0.1388$^{---}$ | -0.0012$^{---}$ | 0.2032$^{*}$ | 0.1626$^{---}$ | 0.2137$^{***}$ | -0.0853$^{---}$ | 0.1283$^{*}$ | | | | | | | | | | | | | | | | | | | | |
| 10 | 0.0229$^{---}$ | 0.2054$^{*}$ | 0.0109$^{---}$ | 0.4402$^{**}$ | -0.0281$^{---}$ | 0.3252$^{**}$ | 0.1212$^{---}$ | -0.0051$^{---}$ | 0.1459$^{---}$ | | | | | | | | | | | | | | | | | | | |
| 11 | 0.0957$^{---}$ | 0.0643$^{---}$ | -0.0619$^{---}$ | 0.2014$^{---}$ | 0.0510$^{---}$ | 0.1657$^{*}$ | -0.0179$^{---}$ | -0.0160$^{---}$ | 0.0223$^{---}$ | -0.0185$^{---}$ | | | | | | | | | | | | | | | | | | |
| 12 | 0.2017$^{***}$ | 0.2920$^{***}$ | 0.1950$^{**}$ | 0.1333$^{---}$ | 0.2788$^{***}$ | 0.1518$^{**}$ | 0.1450$^{**}$ | 0.2507$^{***}$ | 0.1776$^{***}$ | 0.2339$^{**}$ | 0.2343$^{***}$ | | | | | | | | | | | | | | | | | |
| 13 | 0.6071$^{***}$ | 0.5692$^{***}$ | 0.5654$^{***}$ | 0.3764$^{**}$ | 0.6729$^{***}$ | 0.4336$^{***}$ | 0.5743$^{***}$ | 0.5964$^{***}$ | 0.5738$^{***}$ | 0.6983$^{***}$ | 0.2508$^{***}$ | 0.5475$^{***}$ | | | | | | | | | | | | | | | | |
| 14 | 0.5803$^{***}$ | 0.5301$^{***}$ | 0.5383$^{***}$ | 0.4206$^{***}$ | 0.6446$^{***}$ | 0.4969$^{***}$ | 0.4987$^{***}$ | 0.5729$^{***}$ | 0.5193$^{***}$ | 0.6136$^{***}$ | 0.2417$^{***}$ | 0.0836$^{***}$ | 0.5679$^{***}$ | | | | | | | | | | | | | | | |
| 15 | 0.7619$^{***}$ | 0.6256$^{***}$ | 0.6957$^{***}$ | 0.7157$^{***}$ | 0.7643$^{***}$ | 0.7227$^{***}$ | 0.6831$^{***}$ | 0.6989$^{***}$ | 0.6962$^{***}$ | 0.7266$^{***}$ | 0.6537$^{***}$ | 0.7685$^{***}$ | 0.7188$^{***}$ | 0.744$^{***}$1 | | | | | | | | | | | | | | |
| 16 | 0.8206$^{***}$ | 0.6332$^{***}$ | 0.7100$^{***}$ | 0.7178$^{***}$ | 0.7745$^{***}$ | 0.8063$^{***}$ | 0.7064$^{***}$ | 0.7360$^{***}$ | 0.7583$^{***}$ | 0.7062$^{***}$ | 0.7332$^{***}$ | 0.7638$^{***}$ | 0.7358$^{***}$ | 0.8152$^{***}$ | 0.2698$^{---}$ | | | | | | | | | | | | | |
| 17 | 0.8120$^{***}$ | 0.7171$^{***}$ | 0.7558$^{***}$ | 0.7874$^{***}$ | 0.7954$^{***}$ | 0.7966$^{***}$ | 0.7320$^{***}$ | 0.7756$^{***}$ | 0.7624$^{***}$ | 0.7827$^{***}$ | 0.7688$^{***}$ | 0.8098$^{***}$ | 0.7661$^{***}$ | 0.8099$^{***}$ | 0.2485$^{**}$ | 0.1735$^{**}$ | | | | | | | | | | | | |
| 18 | 0.8256$^{***}$ | 0.6805$^{***}$ | 0.7678$^{***}$ | 0.7720$^{***}$ | 0.8094$^{***}$ | 0.8010$^{***}$ | 0.7345$^{***}$ | 0.7489$^{***}$ | 0.7648$^{***}$ | 0.7609$^{***}$ | 0.7399$^{***}$ | 0.8250$^{***}$ | 0.7803$^{***}$ | 0.8067$^{***}$ | 0.2600$^{---}$ | 0.1446$^{---}$ | 0.0034$^{---}$ | | | | | | | | | | | |
| 19 | 0.4289$^{***}$ | 0.2707$^{***}$ | 0.2870$^{***}$ | 0.2526$^{***}$ | 0.4340$^{***}$ | 0.3812$^{***}$ | 0.2511$^{***}$ | 0.4141$^{***}$ | 0.2900$^{***}$ | 0.3867$^{***}$ | 0.3606$^{***}$ | 0.4701$^{***}$ | 0.2644$^{***}$ | 0.4648$^{***}$ | 0.5047$^{***}$ | 0.4236$^{***}$ | 0.5490$^{***}$ | 0.5431$^{***}$ | | | | | | | | | | |
| 20 | 0.5137$^{***}$ | 0.3037$^{***}$ | 0.3687$^{***}$ | 0.3985$^{***}$ | 0.4847$^{***}$ | 0.5440$^{***}$ | 0.3934$^{***}$ | 0.4339$^{***}$ | 0.4517$^{***}$ | 0.4165$^{***}$ | 0.5128$^{***}$ | 0.5457$^{***}$ | 0.3968$^{***}$ | 0.6477$^{***}$ | 0.5883$^{***}$ | 0.5328$^{***}$ | 0.6267$^{***}$ | 0.6167$^{***}$ | 0.1757$^{***}$ | | | | | | | | | |
| 21 | 0.5963$^{***}$ | 0.3892$^{***}$ | 0.4553$^{***}$ | 0.4513$^{***}$ | 0.5264$^{***}$ | 0.5536$^{***}$ | 0.4783$^{***}$ | 0.4209$^{***}$ | 0.5060$^{***}$ | 0.4744$^{***}$ | 0.5255$^{***}$ | 0.6204$^{***}$ | 0.4385$^{***}$ | 0.6780$^{***}$ | 0.7319$^{***}$ | 0.7210$^{***}$ | 0.7624$^{***}$ | 0.7534$^{***}$ | 0.2943$^{***}$ | 0.2744$^{***}$ | | | | | | | | |
| 23 | 0.5211$^{***}$ | 0.2946$^{***}$ | 0.3606$^{***}$ | 0.3809$^{***}$ | 0.4738$^{***}$ | 0.4876$^{***}$ | 0.3848$^{***}$ | 0.4517$^{***}$ | 0.4165$^{***}$ | 0.3699$^{***}$ | 0.4590$^{***}$ | 0.5243$^{***}$ | 0.3613$^{***}$ | 0.5577$^{***}$ | 0.4928$^{***}$ | 0.4523$^{***}$ | 0.5566$^{***}$ | 0.4971$^{***}$ | 0.3065$^{***}$ | 0.2952$^{***}$ | 0.2991$^{***}$ | | | | | | | |
| 24 | 0.5516$^{***}$ | 0.3312$^{***}$ | 0.3788$^{***}$ | 0.4738$^{***}$ | 0.5259$^{***}$ | 0.5397$^{***}$ | 0.4396$^{***}$ | 0.5058$^{***}$ | 0.4618$^{***}$ | 0.4097$^{***}$ | 0.5027$^{***}$ | 0.5862$^{***}$ | 0.4072$^{***}$ | 0.6091$^{***}$ | 0.3811$^{***}$ | 0.4426$^{***}$ | 0.5064$^{***}$ | 0.4448$^{***}$ | 0.3900$^{***}$ | 0.4057$^{***}$ | 0.4650$^{***}$ | 0.1105$^{***}$ | | | | | | |
| 25 | 0.2643$^{***}$ | 0.2052$^{***}$ | 0.1821$^{***}$ | 0.2160$^{***}$ | 0.1868$^{***}$ | 0.2292$^{***}$ | 0.1607$^{***}$ | 0.1751$^{***}$ | 0.1934$^{***}$ | 0.1548$^{**}$ | 0.2327$^{***}$ | 0.3783$^{***}$ | 0.1867$^{***}$ | 0.4200$^{***}$ | 0.4002$^{***}$ | 0.4377$^{***}$ | 0.5364$^{***}$ | 0.4451$^{***}$ | 0.2484$^{***}$ | 0.2791$^{***}$ | 0.2605$^{***}$ | 0.3261$^{***}$ | 0.3779$^{***}$ | | | | | |
| 26 | 0.7356$^{***}$ | 0.5517$^{***}$ | 0.6149$^{***}$ | 0.5679$^{***}$ | 0.7027$^{***}$ | 0.6381$^{***}$ | 0.6054$^{***}$ | 0.6375$^{***}$ | 0.6427$^{***}$ | 0.6453$^{***}$ | 0.6210$^{***}$ | 0.6560$^{***}$ | 0.6060$^{***}$ | 0.6890$^{***}$ | 0.6835$^{***}$ | 0.6396$^{***}$ | 0.7352$^{***}$ | 0.7006$^{***}$ | 0.5477$^{***}$ | 0.4594$^{***}$ | 0.5406$^{***}$ | 0.3866$^{***}$ | 0.4692$^{***}$ | 0.4209$^{***}$ | | | | |
| 27 | 0.7956$^{***}$ | 0.5140$^{***}$ | 0.6602$^{***}$ | 0.6776$^{***}$ | 0.7773$^{***}$ | 0.7722$^{***}$ | 0.6786$^{***}$ | 0.7244$^{***}$ | 0.7221$^{***}$ | 0.7032$^{***}$ | 0.7048$^{***}$ | 0.7746$^{***}$ | 0.6925$^{***}$ | 0.7973$^{***}$ | 0.6915$^{***}$ | 0.5616$^{***}$ | 0.7256$^{***}$ | 0.6646$^{***}$ | 0.4345$^{***}$ | 0.5200$^{***}$ | 0.6744$^{***}$ | 0.2359$^{***}$ | 0.4108$^{***}$ | 0.4527$^{***}$ | 0.4890$^{***}$ | | | |
| 28 | 0.6548$^{***}$ | 0.4425$^{***}$ | 0.5058$^{***}$ | 0.5100$^{***}$ | 0.5961$^{***}$ | 0.5633$^{***}$ | 0.5038$^{***}$ | 0.5315$^{***}$ | 0.5336$^{***}$ | 0.5448$^{***}$ | 0.4667$^{***}$ | 0.6546$^{***}$ | 0.5463$^{***}$ | 0.6863$^{***}$ | 0.6663$^{***}$ | 0.5528$^{***}$ | 0.7187$^{***}$ | 0.6768$^{***}$ | 0.3011$^{***}$ | 0.5182$^{***}$ | 0.6146$^{***}$ | 0.3639$^{***}$ | 0.4739$^{***}$ | 0.2275$^{***}$ | 0.5915$^{***}$ | 0.4494$^{***}$ | | |
| 29 | 0.4572$^{***}$ | 0.2619$^{***}$ | 0.2777$^{***}$ | 0.3268$^{***}$ | 0.4165$^{***}$ | 0.3547$^{**}$ | 0.3228$^{***}$ | 0.3376$^{***}$ | 0.3196$^{***}$ | 0.3662$^{***}$ | 0.2997$^{***}$ | 0.5306$^{***}$ | 0.3439$^{***}$ | 0.5896$^{***}$ | 0.6580$^{***}$ | 0.5949$^{***}$ | 0.7426$^{***}$ | 0.7095$^{***}$ | 0.2658$^{***}$ | 0.3729$^{***}$ | 0.4125$^{***}$ | 0.3370$^{***}$ | 0.4543$^{***}$ | 0.1853$^{***}$ | 0.5429$^{***}$ | 0.4966$^{***}$ | 0.1694$^{---}$ | |
| 30 | 0.5781$^{***}$ | 0.3939$^{***}$ | 0.4414$^{***}$ | 0.3985$^{***}$ | 0.5031$^{***}$ | 0.4934$^{***}$ | 0.4123$^{***}$ | 0.4725$^{***}$ | 0.4470$^{***}$ | 0.4545$^{***}$ | 0.4752$^{***}$ | 0.5627$^{***}$ | 0.4324$^{***}$ | 0.5961$^{***}$ | 0.6058$^{***}$ | 0.5664$^{***}$ | 0.6755$^{***}$ | 0.6046$^{***}$ | 0.3100$^{***}$ | 0.3787$^{***}$ | 0.3630$^{***}$ | 0.2335$^{***}$ | 0.4110$^{***}$ | 0.2743$^{***}$ | 0.4330$^{***}$ | 0.2141$^{***}$ | 0.1498$^{**}$ | 0.2450$^{***}$ |
